# Evaluating Health Co-Benefits of Climate Change Mitigation in Urban Mobility

**DOI:** 10.3390/ijerph15050880

**Published:** 2018-04-28

**Authors:** Brigitte Wolkinger, Willi Haas, Gabriel Bachner, Ulli Weisz, Karl W. Steininger, Hans-Peter Hutter, Jennifer Delcour, Robert Griebler, Bernhard Mittelbach, Philipp Maier, Raphael Reifeltshammer

**Affiliations:** 1 Wegener Center for Climate and Global Change, University of Graz, Brandhofgasse 5, A-8010 Graz, Austria; gabriel.bachner@uni-graz.at (G.B.); karl.steininger@uni-graz.at (K.W.S.); mittelbach@gmx.at (B.M.); 2 Institute of Social Ecology, Alpen-Adria University Klagenfurt, Schottenfeldgasse 29, A-1070 Vienna, Austria; willi.haas@boku.ac.at (W.H.); ulli.weisz@boku.ac.at (U.W.); phqmaier@gmail.com (P.M.); 3 Institute of Social Ecology, University of Natural Resources and Life Sciences, Vienna, Schottenfeldgasse 29, A-1070 Vienna, Austria; 4 Department of Economics, University of Graz, Universitaetsstrasse 15, A-8010 Graz, Austria; 5 Department of Environmental Health, Center for Public Health, Medical University of Vienna, Spitalgasse 23, A-1090 Vienna, Austria; hans-peter.hutter@meduniwien.ac.at; 6 Austrian Public Health Institute (Gesundheit Österreich GmbH), Stubenring 6, A-1010 Vienna, Austria; jennifer.delcour@goeg.at (J.D.); robert.griebler@goeg.at (R.G.); 7 Institute of Internal Combustion Engines and Thermodynamics, Graz University of Technology, Inffeldgasse 19, A-8010 Graz, Austria; reifeltshammer@ivt.tugraz.at

**Keywords:** urban mobility, health co-benefits, physical activity, air pollution, climate change mitigation, interdisciplinary approach

## Abstract

There is growing recognition that implementation of low-carbon policies in urban passenger transport has near-term health co-benefits through increased physical activity and improved air quality. Nevertheless, co-benefits and related cost reductions are often not taken into account in decision processes, likely because they are not easy to capture. In an interdisciplinary multi-model approach we address this gap, investigating the co-benefits resulting from increased physical activity and improved air quality due to climate mitigation policies for three urban areas. Additionally we take a (macro-)economic perspective, since that is the ultimate interest of policy-makers. Methodologically, we link a transport modelling tool, a transport emission model, an emission dispersion model, a health model and a macroeconomic Computable General Equilibrium (CGE) model to analyze three climate change mitigation scenarios. We show that higher levels of physical exercise and reduced exposure to pollutants due to mitigation measures substantially decrease morbidity and mortality. Expenditures are mainly born by the public sector but are mostly offset by the emerging co-benefits. Our macroeconomic results indicate a strong positive welfare effect, yet with slightly negative GDP and employment effects. We conclude that considering economic co-benefits of climate change mitigation policies in urban mobility can be put forward as a forceful argument for policy makers to take action.

## 1. Introduction

Previous studies have shown that implementation of low-carbon policies in the transport sector collaterally increases public health through increased physical activity and improved air quality, called health co-benefits, e.g., [1,2,3,4,5,6]. Yet, with respect to political decision processes, it seems as if these additional benefits (effectively implying policy cost reductions) are not taken into account and the narrowly defined implementation costs of mitigation measures are the prevailing criteria for decision makers.

Globally the transport sector accounts for 14% of the direct greenhouse gas (GHG) emissions (CO_2_equ in 2010) [7]. In Austria this sector is one of the largest emitters of GHG emissions (accounting for 29% of CO_2_equ emissions in 2016) and the sector with the largest increase since 1990 (at 67%) [8]. Nitrogen oxides and particulate matter (PM) impact health adversely, especially in urban areas with specific topographic and climatic constellations. Analyzing the conditions in Austria more closely, we find that in 2017 the number of days with PM_10_ exceeding the threshold level of 50 µg/m^3^ (daily average) were highest in Graz with 49 days, Linz with 25 and Vienna with 20 days [9]. Thus, for modelling health co-benefits of climate change strategies we focus on urban transport for these three largest Austrian cities. Furthermore, the mitigation potential of switching from motorized individual transport to public transport, bicycling or walking is large in urban areas due to available infrastructure and short distances between key locations of everyday life.

Recent studies have investigated the impacts of specific climate change strategies in urban transport and model the corresponding health effects (for an overview see [10,11,12]). The reviews show that most of the studies focus on active transport (biking and walking) [13,14], only some include low emission vehicles [15] and few include the use of public transport [2,3,4,16]. The health effects analyzed refer to changes in physical activity and air pollution; traffic injuries were less often evaluated. In this analysis, we evaluate co-benefits of increased physical activity and improved air quality by applying an interdisciplinary multi-model approach linking a transport modelling tool, a transport emission model, an emission dispersion model for air pollution impacts and a health model. We build on previous work [6,15] that follows a typical procedure from baseline, impact assessment, valuation and sensitivity analysis [17].

Xia et al. [12] address a shortcoming of recent studies that economic valuation of benefits is still at an early stage, focusing only on a specific transport mode (e.g., HEAT Tool of the WHO for cycling [18]) or a specific cost factor like fuel savings (e.g., [14]). A macroeconomic approach considering implementation costs and health benefits of transport policies has only been applied for selected measures and not for the overall urban passenger transport system (e.g., [19]). In a similar vein, Koegh-Brown et al. [20] and Jensen et al. [21] model the implementation of a road pricing tax in order to achieve specific emission reduction targets and technological mitigation in separate scenarios. To the authors’ best knowledge, a comprehensive and detailed macroeconomic assessment of climate change policies as it is presented here, has not been carried out until now. The role of public investments especially has thus far been neglected, but is included here.

We further enhance recent studies in different ways: first, we investigate health impacts for holistic passenger transport scenarios including domestic and commuter transport and considering changes in infrastructures, regulations, pricing and technologies. Three different scenarios are formulated and modelled for this purpose: the ‘Green Mobility’ scenario is based on the urban parliaments’ targets for modal share of trips, the ‘Green Exercise’ scenario simulates a change in mobility behavior beyond the politically-accepted urban targets for the modal share and the ‘Zero Emission’ scenario corresponds to the Green Exercise scenario with all motorized trips conducted with electric energy.

Second, we provide an economic assessment of the implementation (investment and operating costs) of all policies (within the three scenarios) and of corresponding health effects, which both are implemented in a macroeconomic Computable General Equilibrium (CGE) model, calculating impacts on GDP, employment, welfare, relative prices and tax revenues.

Third, to be policy relevant, we start from the climate targets the urban parliaments have set for the modal share in 2020/2025 for urban mobility, i.e., current politically accepted plans, thereby addressing the critique by Xia et al. [12] that recent studies do not base their scenarios on local conditions and future plans of local authorities.

Our results help policy makers to base their decisions not only on public and private investment and operating costs but include information on reduced public and private health costs and reduced productivity losses due to changes in morbidity and mortality and macroeconomic effects. This could convince policy makers to take decisions necessary to fulfill their GHG emission reduction targets.

## 2. Methods

**Figure 1:**
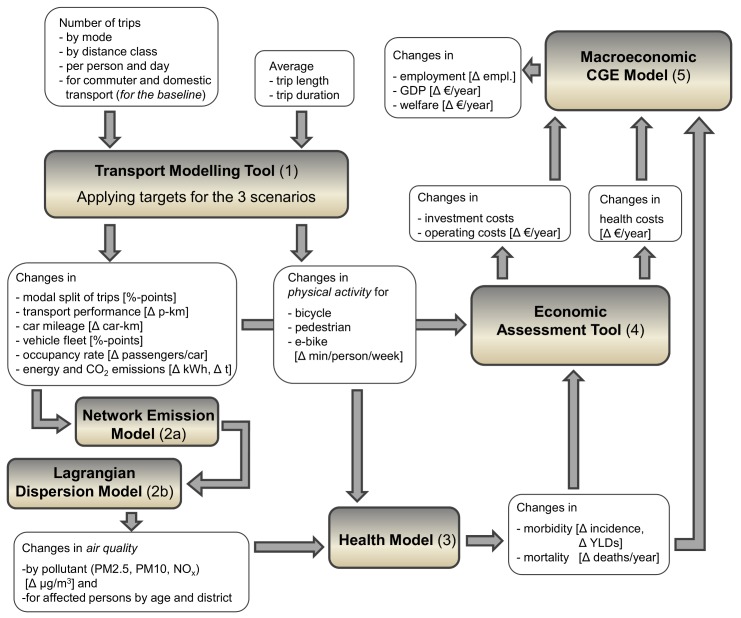
Interdisciplinary multi-model approach for calculating climate change effects and health co-benefits.

For modelling the co-benefits for three types of scenarios in three Austrian cities, an interdisciplinary approach combining different model types has been applied. In general, we choose a comparative static approach. This means that the effects of any policy intervention on the transport system are compared to a case without such an intervention (*ceteris paribus*). We choose the year 2010 as a point of reference (“baseline”) and calculate changes in transport performance and mileage, greenhouse gas emissions and air pollutants relative to the baseline. Put differently, we assume that the policy intervention has already been achieved in 2010 and compare this hypothetical state to the baseline. Starting with the modal share targets of the local parliaments for the three cities, the Transport Modelling Tool (1, cf. Figure 1) models changes in transport performance and mileage per transport mode and corresponding changes in energy use and CO_2_equ emissions. This output serves as an input for the transport Network Emission Model (NEMO) (2a), which calculates road transport emissions (pollutants) by applying a top-down approach for assigning transport parameters to the traffic grid. This output is then used in a highly resolved Lagrangian Dispersion Model (2b) to model changes in emissions and concentration levels of pollutants for different health-related pollutants while using meteorological data (e.g., wind) and detailed fleet parameters (cf. Figure 1).

Matching these data with data on population distribution yields the number of persons per district that are exposed to these changes in air quality. A second output of the Transport Modelling Tool refers to increased physical activity due to changes in modal share of trips (additional trips by bike, e-bike or foot), both affecting health. Based on changes of exposure of persons to emissions, relative risks obtained from literature (meta study) and changes in physical activity, the Health Model (3) calculates changes in morbidity and mortality for different diseases (i.e., cardiovascular diseases, lung cancer, respiratory diseases and myocardial infarction). Thus two input arrows from two different models enter into the Health Model as can be seen in Figure 1. This information is used for the Economic Assessment Tool (4) to calculate changes in private and public health costs as well as investment and operating costs for corresponding mitigation policies and in the macroeconomic CGE model (5). Finally, this CGE model calculates changes in employment, GDP and welfare using data of changed investment and operating costs and interrelated sectors as well as changes in health costs.

### 2.1. Mobility Scenarios

We develop three scenarios for changes in urban passenger transport, based on the transport action plans of the local governments of Graz, Linz and Vienna, which define targets for the modal share of trips for domestic and commuter transport [22,23,24,25] and partly corresponding policy strategies. Additionally, the action plans supply target levels for the occupation rate of car transport.

In the first scenario, ‘Green Mobility’ (GM), we assume that the politically-determined targets for the modal share of trips for the years 2020/2025 are almost achieved. Since we follow a comparative static approach, future changes in population and transport demand are not considered. The relevant policy strategies of the action plans comprise the improvement of pedestrian and bicycle infrastructure and service (environmental/pedestrian zones, restricted access for cars in the inner city), improvement of public transport and service as well as parking regulations.

In the second scenario ‘Green Exercise’ (GE) we assume a change in mobility behavior beyond the policy targets. This can be achieved by additional measures to promote active mobility (pedestrian and bike) and an expansion of access restrictions for cars in city centers combined with an expansion of public transport supply. In this scenario, domestic transport by cars is drastically reduced and commuter transport requires introduction of further incentives, such as price reductions for public transport.

**Figure 2:**
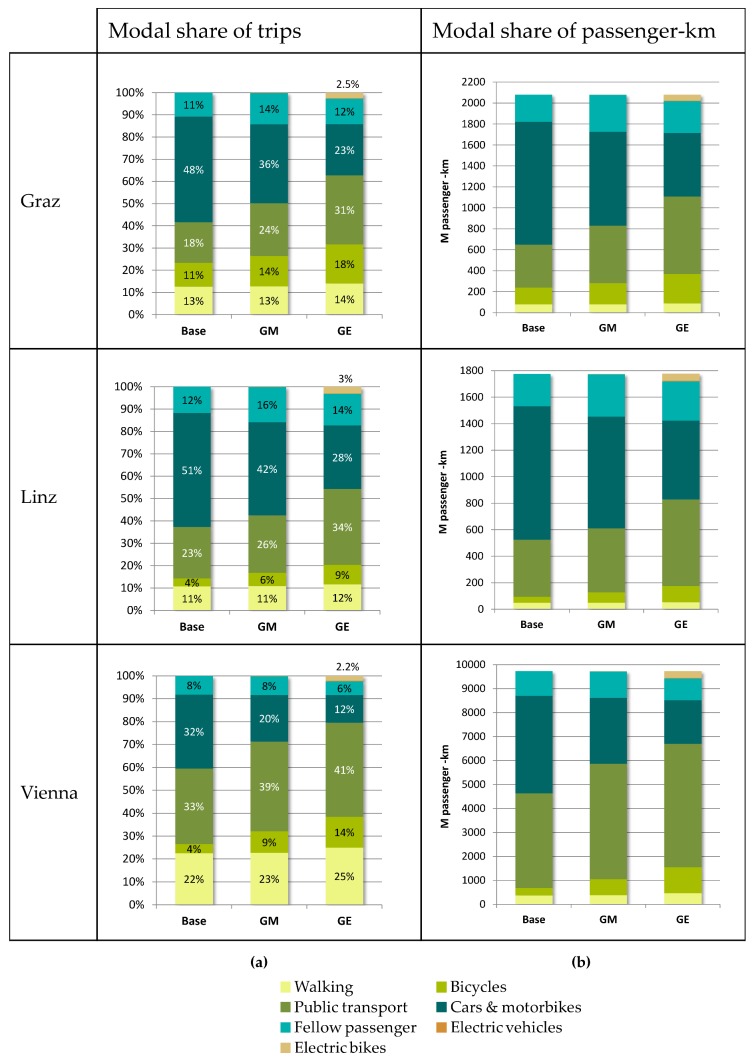
Modal share of trips [%] (**a**) and transport performance (passenger-km) (**b**) for the three cities for the Baseline (Base), Green Mobility (GM) and Green Exercise (GE) Scenario.

For example, the share of car trips (domestic and commuters) in Vienna is reduced from 40% to 28% in the Green Mobility scenario and to 18% in the Green Exercise scenario. In turn the share of bike trips increases from 4% to 9% in 2010 and 14%, respectively compared to the baseline (see Figure 2).

The focus of the third scenario ‘Zero Emissions’ (ZE) is long term and can be seen as a contribution towards achieving the 2 °C target that requires a substantial reduction of GHG emissions to near zero as mentioned in the IPCC’s “Summary for policymakers” [26]. To meet this requirement, we assume the same modal shares as in the Green Exercise scenario but replace the remaining vehicle- or passenger-kilometers, driven by conventional combustion engines, with electric cars and electric public transport.

The left-hand side panels of Figure 2 show the changes in the modal share of trips (share of trips in %) for each city, for the baseline, Green Mobility (GM) and Green Exercise (GE) scenarios, the latter of which corresponds to the Zero Emission (ZE) scenario in terms of changes in transport performance (for both domestic and commuter transport). On the right-hand side, the modal share of transport performance is displayed (passenger-km).

### 2.2. Modelling Changes in Transportation, Physical Activity and Air Pollution

Applying the Transport Modelling Tool (1), we derive changes in transport performance and mileage by scenario. The underlying data for this tool is based on local and regional transport surveys for working days and is comprised of the following baseline parameters for each city:

- Number of trips by category of trip length and mode (#)
- Modal Share (%)
- Number of trips per person and day (#/d/p)
- Average trip length by transport mode (km)
- Average trip duration by transport mode (h)

These input data are entered into the Transport Modelling Tool (1) as shown at the top left in Figure 1. Traffic for weekends and commuter transport are based on the commuter statistics and regional transport surveys [23,27]. The tool is used to reach the overall modal share targets of the scenarios by shifting trips from one (motorized individual transport) to other groups (public transport, bicycle or pedestrian) within a certain distance category. Table 1 illustrates for Vienna for the GM scenario that 65% of all very short car trips (up to 1 km) are shifted to other modes of transport, 8% thereof to pedestrian, 83% to bicycle, 7% to public transport and 1% to electric car and electric bike (pedelec) trips each. For each trip length category, the values are calibrated to meet the overall target shares of number of trips by mode as defined in the scenarios (Figure 2). Additionally, increased vehicle occupation rates for domestic and commuter transport (determined by local parliaments) are applied which further reduce overall absolute mileage.

**Figure 3:**
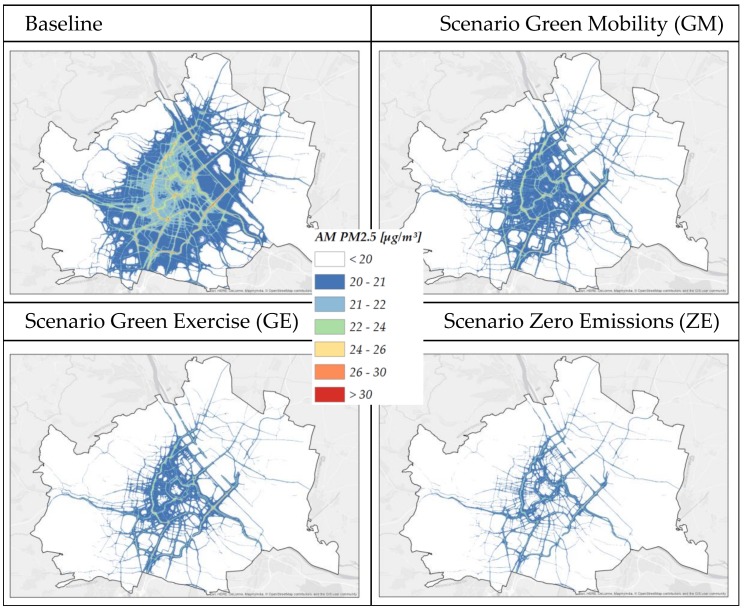
Annual mean concentrations of PM_2.5_ (μg/m^3^) for Vienna (baseline and scenarios).

The resulting changes in transport performance and mileage by transport mode as specified by the Transport Modelling Tool (1) enter into the transport Network Emission Model (NEMO) [28] (2a in Figure 1) which calculates road transport emissions (exhaust and non-exhaust) using detailed data on fleet composition (category, fuel types, emission classes etc.) and on road network (section lengths, gradients). These calculated road transport emissions, combined with meteorological data, comprise the input for the Lagrangian Dispersion Model GRAL [29], which is used to model concentration levels of pollutants for each scenario [30] (2b in Figure 1). Different diurnal and seasonal cycles are used to consider variability of pollutant sources. Changes in the annual mean of specific pollutants are calculated and matched with population data on the district level to determine the number of people affected by a specific change in exposure to specific pollutants. Figure 3 illustrates spatially-explicit modelled concentration levels for the city of Vienna at high resolution (10 m × 10 m). Air pollution (annual mean for PM_2.5_ concentration) is exemplified by different colors for different concentration levels of pollutants for the baseline case and the three scenarios. Detailed concentration level maps for all cities and considered pollutants are given in Figures S3–S5 in the Supplementary Material.

In addition, we estimated health co-benefits from changes in the physical activity of pedestrians, bicyclists and e-bike users. These health effects are usually presented in relation to the weekly number of minutes people do additional exercise. In determining such effects we therefore need to derive both the number of people with increased activity and their extra minutes in active motion, both from our Transport Modelling Tool. By assuming average velocities for the different active modes we can convert the extra mileage of walking and biking into extra minutes. For example, for the Green Exercise scenario in Vienna there are 250,000 people using a bike for their trips instead of a car, yielding an additional 214 minutes of biking per week. Detailed results for all cities are given in Section 3.1.

### 2.3. Modelling Health Effects from Changes in Modal Shift

#### 2.3.1. Physical Activity

Based on changes in physical activity (Δ min/person/week) (see results of the Transport Modelling Tool, (1) in Figure 1) we can estimate the related changes in mortality (by the Health Model, 3). In the case of physical activity we do not need to calculate all three scenarios, since the scenario Zero Emissions (ZE) only replaces the combustion-engine-driven mobility in the Green Exercise (GE) scenario by electric vehicles, which does not alter the level of physical activity. The health impacts of increased physical activity are quantified by modelling the relationship between these factors and mortality, based on existing meta-analytical estimates. The functional relationship between Metabolic Equivalent of Task (MET) hours per week and the relative risk for all-cause mortality was estimated based on available meta-analyses [31,32,33,34] (MET is an equivalent of physical activity. It is in units of basic metabolic rate [35]). Accordingly, a more physically active person’s relative risk of death (RR; defined as the ratio between the risk of an exposed person to the risk of another person not or less exposed) is lower than that of another person with lower activity levels. RR and Hazard Risks (HR) are similar indicators, albeit with small conceptual differences related to time. Hazard ratio provides instantaneous risk at a particular time and relative risk pertains to cumulative risk over a span of time [36]. Based on individual HR we calculated HR for atraumatic mortality for the affected group for each city, considering the actual changes in activity levels introduced by the scenarios. The atraumatic mortality includes all mortality causes except injuries (code: A00-R99; World Health Organisation International Classification of Diseases, ICD-10 [37]).

With the HR as shown in Table 2, in combination with population [38] and cause of death statistics [39], we calculate the potential changes in mortality due to the discussed changes in urban mobility for 2010. For those persons engaged in active mobility in each scenario, the number of deaths was determined from cause of death statistics, in proportion to the size of the group, to calculate the mortality rate before the additional exercise. Applying the HR reduces mortality accordingly, as the design of the study implies that the group would have already been engaged in such additional active mobility by the year 2010, with its corresponding health benefits e.g., reduced mortality risk. Data on Years of Life Lost (YLL), YLL is an indicator for lost years due to premature mortality, were obtained from the Institute for Health Metrics and Evaluation database [40], but only for Austria as a whole. This necessitated calculation of a YLL per death ratio, which was applied for the three target cities to calculate the amount of YLL per city.

#### 2.3.2. Air pollution

Based on the reduced exposure of the general public to air pollution, we calculate reduced morbidity and mortality figures for each city and scenario (in this case, the ZE scenario reduces health-relevant pollutants beyond the reductions of the GE scenario). We can perform this calculation similarly for all three most commonly used health-relevant air pollutants (PM_10_, PM_2.5_ and NO_2_) but it bears consideration that the reduced mortality for each of the pollutants cannot simply be summed up, as health effects are overlapping, meaning that a reduced case of death due to reduced NO_2_ cannot be saved a second time via reduced PM_10_. Since these pollutants affect the same group and usually occur in combination, we can therefore only consider the strongest result for further calculations.

The health impacts of a reduction in air pollution were quantified by modelling the relationship between these factors and mortality, based on meta-analytical estimates displayed in Table 3. For coronary events and lung cancer, Hazard Risk (HR) indicators are applied. Relative risk values for the diseases have been obtained by carrying out a systematic review including meta-analysis of large, international epidemiological studies [41,42,43,44,45,46].

Based on the exposure of persons to the included pollutants (NO_2_, PM_2.5_ and PM_10_) and the RR and HR, new values for “Atraumatic mortality (A00-R99)”, “Cardio-vascular mortality (I00-I99)”, “Respiratory mortality (J00-J99)”, “Myocardial infarction (I21-I22)” and “Lung cancer (C33-C34)” (according to the ICD-10 classification) were calculated. Data from the National Causes of Death Statistics [39], the Austrian Cancer Registry [47] and the Austrian Hospital Admission Statistics [48] is used. The myocardial infarction incidence is estimated by combining data from the National Causes of Death Statistics [39] and from the Austrian Hospital Admission Statistics [48]. Data on Years of Life Lost (YLL) and Years lived with Disability (YLD) were obtained from the Institute for Health Metrics and Evaluation database [40] for all mentioned outcomes.

Since the cities’ entire population is affected, we obtained the relevant mortality and incidence data from statistics mentioned above for the baseline. The resulting actual risks can be further altered by applying the RR as displayed in Table 3 to obtain a new number for the different risks and finally, to show the reduced death cases or incidences. For a more detailed explanation of the Health Model see chapter S6 in the Supplementary Material.

### 2.4. Economic Assessment

Based on the results of the Transport Modelling Tool (1), the Lagrangian Dispersion Model (2b) and the Health Model (3), changes in costs and benefits (i.e., saved costs) relative to the baseline were assessed by the Economic Assessment Tool (4).

#### 2.4.1. Investment Costs and Operating Costs

As we are interested in a comprehensive evaluation of health co-benefits of climate change mitigation measures, we also have to account for the associated costs of these measures. The costs are either one time investment costs (for the implementation of e.g., charging infrastructure) or recurring operating costs. In addition we account for who is bearing the different costs; either the public sector or private households.

In the first step, we calculate all the investment and operating costs for the required mitigation measures. Implementation costs and corresponding operating costs are calculated for the investments throughout the total planning period by OENACE sectors [49] and investor (private/public) and broken down on an annual basis by applying typical amortization rates. OENACE is the Austrian version of the Statistical Classification of Economic Activities in the European Community (called NACE). The NACE version used is revision 2. Additionally, changes in private expenditures (as a part of operating costs) due to a shift from motorized individual transport to pedestrian or bike are calculated based on the Austrian Household Budget Survey 2009/2010 [50]. Household expenditures are combined with data on transport performance by mode [51] in order to calculate cost factors per saved car-kilometer (see chapter S5 of the Supplementary Material for an exemplified calculation).

#### 2.4.2. Health Costs and Benefits

For evaluating changes in health costs we apply the cost-of-illness approach, which distinguishes between direct and indirect cost components [52,53,54] and intangible costs. Direct costs refer to costs for medical procedures and services for treatment and care of a disease. They can further be divided into direct medical and direct non-medical costs (cf. Le et al. [55]). The former include medical treatment (in hospital or outside) and medicine while the latter include costs of transportation of patients and family members, costs of accommodation during visits etc. The latter costs are mainly borne by households privately and could not be appraised due to lack of data. Indirect costs refer to productivity losses due to morbidity and mortality. Intangible costs are controversial as they refer to costs from e.g., pain, anxiety or the hypothetical costs for the value of life lost. While former studies value premature death by means of the value of statistical life (VSL) there is growing recognition that it is more meaningful to value changes in life expectancy and to apply the value of life year (VOLY), especially for air pollution, which shortens everyone’s life to some extent [56,57]. Desaigues et al. [58] conducted a large contingent valuation study for European countries and recommend a value of 40,000 € per life year lost due to premature death (Years of Life Lost, YLL) and disease (Years Lived with Disability, YLD) expressing the willingness to pay for extending one’s life expectancy. We multiply a VOLY of 43,000 € which is adjusted by the Austrian CPI (consumer price index) value of 7.9% [59] for the base year 2010 by changes in YLL and YLD due to increased physical activity and improved air quality. This value is a conservative one as other European studies use higher values e.g., 50,000 € from ExternE [57] or 60,000 € from the HEIMTSA study [60]. In a sensitivity analysis we calculate the economic effects for a higher VOLY and compare them to the VSL results (applying a value of 1,650,000 € [60] for changes in death cases).

As the list of cost components in relation to different diseases is long, in practice there are limits to what can be measured and valued due to lack of consistent data of the health care system. In the following we describe which cost components have been valued in the underlying study.

The calculations were based on the results of the Health Model (3) which models changes in morbidity for cardiovascular diseases, respiratory diseases, myocardial infarction, lung cancer and atraumatic mortality. Changes are expressed in incidence, deaths as well as corresponding YLD or YLL. These input data enter into the Economic Assessment Tool (4) as can be seen in Figure 1.

Calculations of changes in direct medical costs are based on the available data for acute stationary stays for 2010 [61]. Absolute numbers for YLD for the base year 2010 represent the years of life living with disability or the state of health within the population for this year. Thus we merge the costs for stationary stays (private and public) with the YLD for the year 2010. With the hence derived costs factor (€/YLD), decreases of YLD per disease produced by the Health Model are valued. As within the Health Model YLD changes can only be calculated for changed air quality, but not for changes in physical activity, effects of the latter are appraised by applying the results of Alt et al. [62]. These authors appraise the national costs of inactivity and the benefits for increased physical activity. They include direct and indirect health cost components and consider increased accidents due to increased activity which partly offset health benefits. Based on the number of active persons and the direct and indirect health costs for the base year derived from Alt et al. [62], a cost factor (of €/active person) is calculated. This factor is multiplied with the changes in the amount of active persons due to a switch from car to pedestrian or bike (derived from the Transport Modelling Tool) in order to calculate changes due to increased physical activity. Expenditures on medicine are appraised from the literature, taking the lower bound of about 5% due to lack of data for Austria (range between 5–35% depending on the disease, cf. e.g., Brown et al. [63].

Changes in productivity of the labor force due to morbidity are calculated by valuing the reduction of sick leaves with the gross median income per working day. This is done by merging incidence numbers with the average number of days with sick leaves per disease and year for the baseline (Table 4) [64].

The gross median income per employee for 2010 was obtained from Statistics Austria (2016) and was divided by 244 (working days) to yield the cost per working day of about 100 €. Cost changes due to reduced mortality per disease category were calculated by applying a human capital approach [65]. Productivity costs due to mortality are thus measured by gross wages lost for the period from the age when death occurs to the age of retirement.

Changes in mortality in the scenarios are only given in absolute terms and not divided by age group. In order to obtain the productive years until the age of retirement and monetize them with the average gross median income, we transfer the current distribution of deaths across age groups for Austria from the Global Burden of Disease Study 2015 [40] to the changes in mortality in the scenarios. Previously lost productive years due to morbidity that can be regained in the scenarios lead to cost savings. In the underlying study, benefits from reduced productivity losses are considered.

### 2.5. The Macroeconomic Model

In a final step we feed the derived costs and benefits into a macroeconomic national Computable General Equilibrium (CGE) model, which is described in Bachner [66]. The model is based on the input-output table of Statistics Austria (2011) and describes the Austrian economy as annual monetary flows across producers, consumers and the government. These flows are in equilibrium in which all markets are cleared simultaneously. Austria is modelled as a small open economy, therefore part of the domestic production is exported. Imports and domestic goods are only partially substitutable [67]. A detailed description of the model type is provided for example by [68].

The model comprises two representative end users (a representative private household and a government) as well as 48 production sectors (see Table A1 in the Appendix). The private household is endowed with the production factors labor and capital, which generate income when supplied to the market. This factor income (wages and capital income) is used by the household to maximize utility, subject to a nested constant elasticity of substitution (CES) function. Production sectors use the supplied production factors and combine them together with other intermediate inputs (i.e., output from other sectors) to produce goods and services, also subject to a nested CES function. The government yields its income from taxation and spends it for public consumption, such as transport infrastructure, schools or the health system.

As all sectors and end users are interconnected (due to the underlying input-output structure), localized interventions in one part of the model (e.g., the mobility sector, or in the household’s or government’s final demand structure) have indirect impacts on all other sectors and end users, leading in turn to overall macroeconomic effects, which are measured as changes in GDP, employment or welfare (relative to the baseline).

The procedure to determine macroeconomic effects is the following: the baseline equilibrium (year 2011) in markets is exogenously shocked, e.g., by changes in transport-related expenditures by households, leading to changes in demanded quantities and consequently to changes in relative prices until a new equilibrium emerges (in which all markets are cleared again). By comparing model variables between the two equilibria (i.e., prior and after the shock), we capture the long-term effects for the whole economy but also for different sectors and end users. Again, note that we follow a comparative static approach.

In the CGE model we account for the direct expenditures necessary for implementation of the policies (as described in Section 2.4.1) as well as for the health benefits (i.e., cost savings) emerging from these policies (as described in Section 2.4.2).

Regarding the direct costs for policy implementation we model (i) changes in private operating costs due to changes in transport behavior (e.g., less demand for fossil fuels and repair services but more demand for electricity), (ii) changes in private investments (e.g., more investment in electric cars and bike, but less in conventional cars), (iii) changes in public operating costs (e.g., higher costs for public transport) and (iv) changes in public investments (e.g., additional costs for civil engineering for railroads).

Health cost savings that have been assessed work through two main channels in the CGE model: First, the changes in sick leaves are translated into changes of labor productivity and second, the changes in health expenditures for the government are reduced (leaving more budgets available for other public good provision). The overall macroeconomic effects are displayed as changes in GDP, welfare, employment rate, relative prices and tax revenues.

## 3. Results

### 3.1. Transport and Environment

If measures are implemented leading to changes in the modal share of trips in line with targets by the local governments, substantial changes in vehicle mileage conducted by car can be expected. In the Green Exercise or Zero Emission scenario (which is equal with respect to changes in transport performance) this leads to a reduction in vehicle mileage of up to 55% relative to the baseline for Vienna (see Table 5 last column). Additionally bus kilometers relative to the baseline will increase up to 35%. For Graz this increase is highest as bus is the predominant transport mode at present, while in Vienna and Linz, electric transport modes are used that can be enhanced.

**Figure 4:**
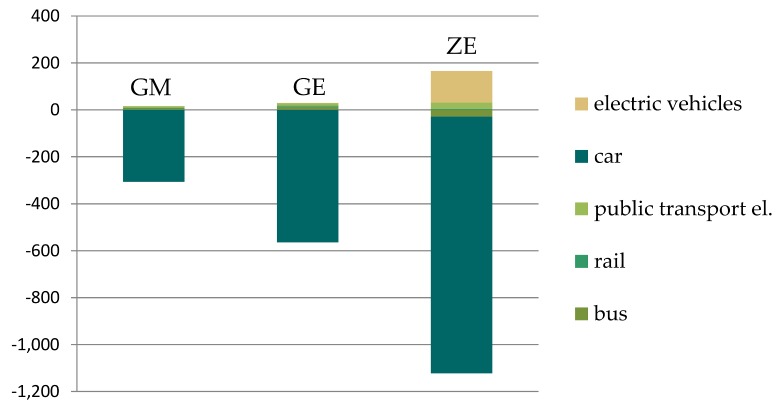
Changes in CO_2_ equivalent emissions (1000 t) for the three scenarios (all urban areas) relative to the baseline.

Correspondingly, these changes lead to substantial reductions in energy use and GHG emissions. Summarizing the climate mitigation impact of all three cities, the targeted trip changes lead to a reduction of about 290,000 t CO_2_equ in the Green Mobility scenario and of almost 530,000 t CO_2_equ in the Green Exercise scenario as displayed in Figure 4. For the Zero Emission scenario the switch from conventional engines to electric engines will lead to a reduction of CO_2_equ of 1,000,000 t relative to the baseline (1,200,000 t in 2010, i.e., 12% of the Austrian emissions in the passenger transport sector). If electric power is produced in a carbon neutral manner, emissions are reduced to zero.

Besides the effects on overall mileage and emissions of CO_2_equ and other pollutants, these changes translate to additional physical activity. Table 6 shows the number of people switching to healthier ways of transportation for each scenario and the additional minutes per person and week. These results enter into the Health Model (3) as displayed in Figure 1.

### 3.2. Health Effects

For the Green Mobility scenario, health results due to increased physical activity and air quality show a prevention of 417 atraumatic deaths due to physical activity and between 3 and 135 cases due to improved air quality depending on the pollutant and the disease. For the Green Exercise and Zero Emission scenario with a higher amount of people using active mobility, 891 deaths are prevented for all cities. The changes due to air quality are higher for the Zero Emission scenario than for the Green Exercise scenario with 284 to 647 prevented death cases, respectively (Table 7).

**Figure 5:**
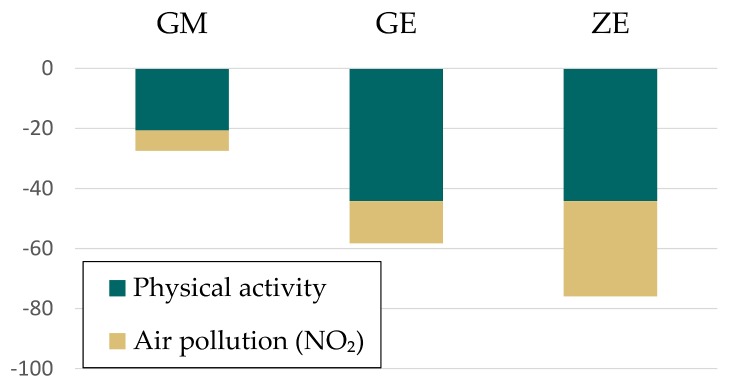
Changes in atraumatic mortality per 100,000 inhabitants due to increased physical activity and cardiovascular disease mortality changes due to NO_2_ decreases.

Reduced atraumatic mortality due to physical activity and cardiovascular disease mortality due to reduced NO_2_ exposure is displayed in relation to 100,000 inhabitants in Figure 5, summarized for all cities. Due to increased physical activity and air quality, decreases in mortality between 27 and 58 per 100,000 inhabitants (GM and GE scenario) can be expected. For the Zero Emission scenario this number is even higher with 76 less cases of death/100,000 inhabitants.

Table 8 gives a more detailed view on disease-specific mortality for the pollutants NO_2_, PM_2.5_ or PM_10_. Changes in YLD which are applied for calculating changes in health costs (Economic Assessment Tool, 4, in Figure 1) are only available for lung cancer and myocardial infarction and are displayed in brackets in corresponding rows.

A reduction of NO_2_ can save 138 to 665 lives (deaths due to cardio-vascular disease and respiratory disease). The effect of PM_10_ reduction can prevent 27 to 60 myocardial infarction (MI) incidence cases and would reduce the YLD by about 0.4 to 0.9 years. Furthermore, the number of avoided lung cancer cases by PM_2.5_ reductions ranges between 23 avoided cases for the GM scenario and 30 for the ZE scenario and would reduce the YLD by about 2.2 to 3.1 years.

**Figure 6:**
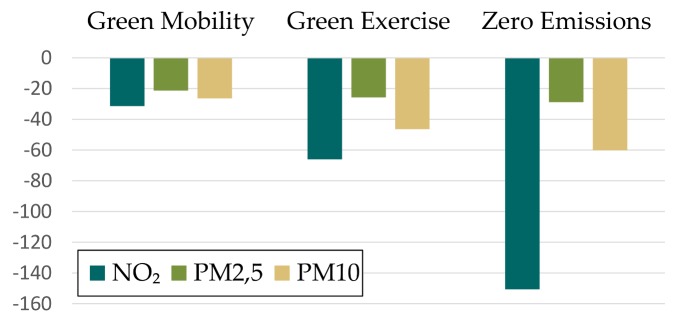
Changes in Disability-Adjusted Life Years (DALYs) per 100,000 inhabitants for the pollutants NO_2_, PM_2.5_ and PM_10_.

Figure 6 shows changes in Disability-Adjusted Life Years (DALYs) which are calculated as the sum of the YLL due to premature mortality in the population and the YLD for people living with the health condition or its consequences [69].

### 3.3. Economic Effects of Mitigation Measures and Co-Benefits

#### 3.3.1. Changes in Implementation Costs, Operating Costs and Household Expenditures

**Figure 7:**
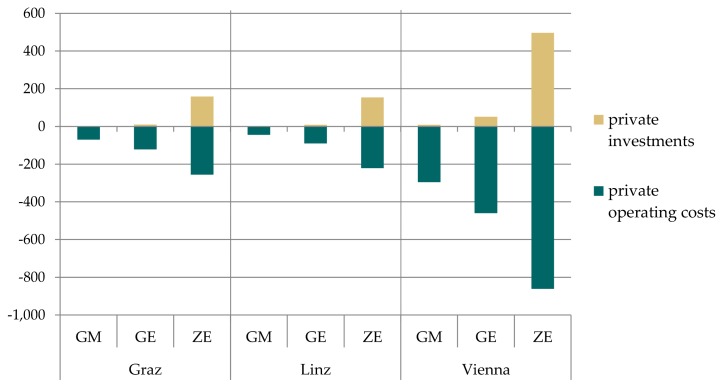
Changes in private investment and operating costs relative to the baseline for all three cities and scenarios (M € p.a.).

Figure 7 shows changes in expenditures for private households due to the shifts from car trips to cycling and walking trips as well as trips conducted by public transport. The savings primarily include operating costs of conventional cars. Considering the total timespan up to 2025, a gradual reduction of new car purchases is considered, leading to additional savings of fixed cost components. Private investment costs are higher for the ZE scenario compared to the other scenarios, as all conventional drives have been shifted to electric vehicles in this scenario and the purchase price for electric vehicles is much higher. Still, there is a net saving for the households in the ZE scenario since private operating costs are reduced to a higher degree.

**Figure 8:**
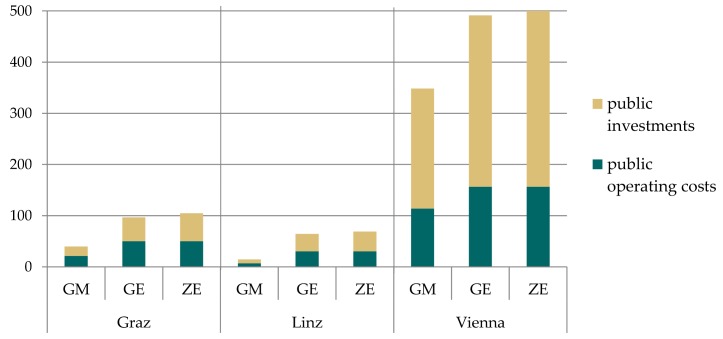
Changes in public investment and operating costs relative to the baseline for all three cities and scenarios (M € p.a.).

For the public sector, additional net costs occur due to investments in public transport, pedestrian and bike lanes and the provision of charging infrastructure for e-mobility in public space (Figure 8).

**Figure 9:**
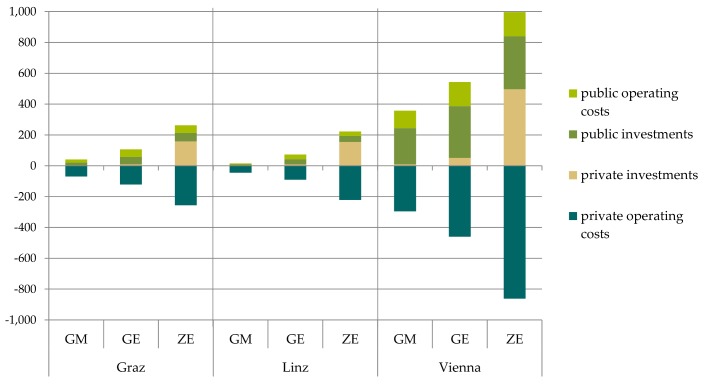
Summary of costs and benefits in private and public investment and operating costs relative to the baseline for all three cities and scenarios (M € p.a.).

Figure 9 contrasts the additional costs with the savings for the different scenarios and cities due to implemented climate change mitigation measures. For the GM and GE scenario savings for private households exceed expenditures from households and the public sector for Graz and Linz. For Vienna costs are slightly higher than savings due to the very ambitious modal share targets and required extension of public transport, bike and pedestrian infrastructure. For the ZE scenario, expenditures slightly exceed savings in all three cities due to additional costs for switching from conventional drives to electric vehicles. Future cost degradation was not included in the calculation which could change the results.

#### 3.3.2. Changes in Health Costs

Table 9 summarizes changes in direct and indirect health costs due to changes in air quality and physical activity for the three Austrian cities and the three scenarios. 

The largest effects are observed in the ZE scenario (total: savings of 19 million € p.a.) with similar monetary effects from the two sources each, changes in air quality (9.8 million €) and changes in physical activity (9.2 million €). In addition, Table 9 shows a range of intangible costs due to increased health and avoided deaths relative to the baseline. Depending on the VOLY unit saved costs account for 350 to 740 million € or 490 to 1026 million € (from GM to ZE scenario). Applying the VSL shows total values which are higher by the factor 2 to 3. Note, that even the most conservative of these valuations for both the GE and ZE scenarios indicate intangible benefits arising from these policies in just these three cities that are equivalent to 0.25% of overall Austrian GDP.

#### 3.3.3. Macroeconomic Effects

The national macroeconomic impacts arise from changes in private and public expenditures for mobility (consumption and investment) and from changes in health expenditures funded by public authorities. Additionally, there are macroeconomic effects due to changes in work absence and morbidity leading to increases in productivity of the labor force. Changes in mortality have not been considered in the CGE model. Note that changes in private consumption are implemented by means of changes in the underlying utility function of the representative household. We thus assume that utility before and after the change in transport demand stays constant, but at different (lower) cost.

**Figure 10:**
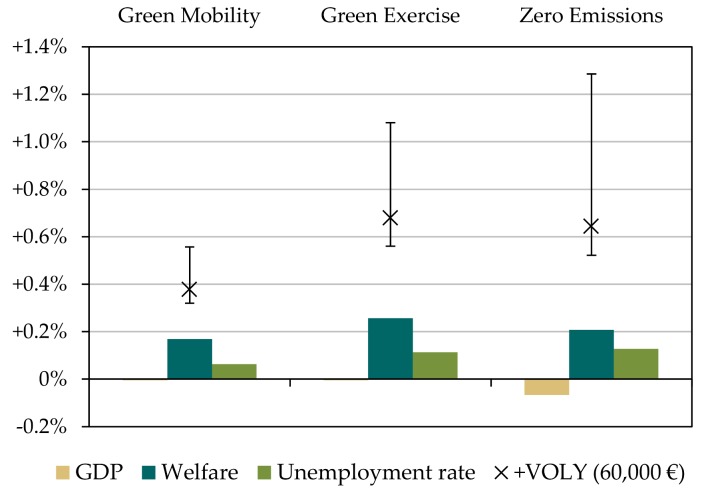
Changes in Gross Domestic Product (GDP), welfare and unemployment for the three scenarios. Error bars indicate welfare effects when additionally accounting for intangible benefits (upper range: using VSL approach with 1.65 million € per life [60]; lower range: using VOLY approach with 43,000 €/VOLY [58]).

Figure 10 summarizes the macroeconomic effects. The most important finding is that for all scenarios there are welfare gains, ranging between +0.15% and +0.25%, with the strongest effect in the Green Exercise scenario. The relatively strong positive welfare effect is a result of changes in private consumption. Private households spend less on mobility, while their utility derived from mobility remains constant. On the macroeconomic level the welfare level thus increases. The effect on GDP, however, is slightly negative and correlates with the effects on the labor market. This is due to a shift of private and public expenditures from relatively labor-intensive goods and services (sale of cars, repair of conventional cars) to capital intensive goods (public transport infrastructure, rolling stock). The unemployment rate thus increases in all scenarios between 0.05% to 0.1% points, causing negative impacts on GDP. This effect is strongest in the ZE scenario, due to the relatively strong decrease in demand for retail and repair of cars with conventional drives as in this scenario all drives have been replaced by electric motors (cars and public transport). When additionally accounting for the intangible benefits as shown in Table 9, the welfare effects are much stronger. Depending on the evaluation method (VOLY or VSL), the welfare effect is between +0.3% (GM, VOLY: 43,000 €) and +1.3% (ZE, VSL: 1.65 million €).

**Figure 11:**
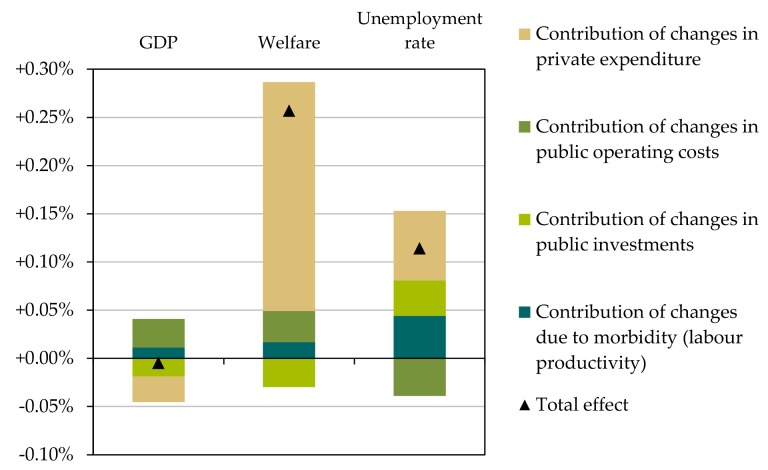
Decomposing the effects of climate mitigation measures on GDP, welfare and employment (illustrative for the Green Exercise scenario).

Decomposing the total macroeconomic changes of GDP, welfare and unemployment shows the contribution for the different cost components private expenditures, public expenditures (investment and operating costs), as well as for changes in labor productivity due to morbidity. Figure 11 shows this decomposition for the Green Exercise scenario.

The overall positive welfare effect (+0.25%) is mainly driven by the decrease in necessary private expenditures, and only to a small extent by changes in public operating costs and morbidity. Shifts in public investment slightly diminish the overall welfare effect, however the total effect is clearly positive. When decomposing the GDP effect, we see that shifts in public operating costs and decreased morbidity enter positively into the GDP effect, while the changes in the composition of the public investment structure and private expenditure structure reduce this effect leading to an overall neutral effect on GDP. Regarding unemployment, we find that the changes in the structure of public and private expenditures and decreased morbidity lead to an increase in the unemployment rate while operating costs (e.g., staff for public transport) decreases unemployment.

### 3.4. Summary of Results

If the city governments of Vienna, Graz and Linz (about 2 million inhabitants) implement their already ambitious plans for passenger transport, as modeled in the Green Mobility scenario, this would lead to a 25% reduction of GHG emissions from passenger transport (minus 0.3 Mt CO_2_equ) and about 550 deaths due to better air quality and more physical exercise.

If city governments were to intensify the measures, especially with regard to health improvements, it would be a worthwhile—although politically challenging—endeavor, as 44% of the GHG emissions (minus 0.5 Mt CO_2_equ) can be reduced. Due to a further reduced risk of death, the number of deaths decreases by 1200 compared to the baseline.

If, as has already been discussed, combustion engines will be replaced by electric engines as in the Zero Emission scenario, further GHG emissions reductions can be achieved. Particulate matter produced by means of abrasion and resuspension remain, while particulates due to combustion disappear, as well as emissions of NO_2_. CO_2_equ emissions disappear completely (reduction of about 1 or 1.2 million t CO_2_equ if electricity is generated carbon-neutrally) and 1500 deaths can be avoided relative to the baseline (displayed separately in Table 10 for air quality and physical activity).

Valuated in monetary terms, health benefits account for up to 11 million € relative to the baseline per year. Additional costs for implementation and operation of public transport and bike or pedestrian facilities are mostly compensated by saved costs for motorized individual transport. For the Green Mobility and Green Exercise Scenario, costs are mainly borne by the public sector while benefits are generated by private households. For the Zero Emission scenario, additional costs for e-cars are borne by private households who are, however, compensated by benefits due to the switch from combustion to electric engines. If intangible costs (VSL) are included health co-benefits are substantially higher by a factor around 100.

Macroeconomic effects on gross domestic product (GDP) are weakly negative and strongly affected by a slightly lowered employment rate and by reduced health spending. However, for all scenarios, the increase in welfare is between +0.2% and +0.3%. The relatively strong positive welfare effect is mainly due to an increase in free disposable income, as private households can still meet their mobility needs with lower expenditures.

**Figure 12:**
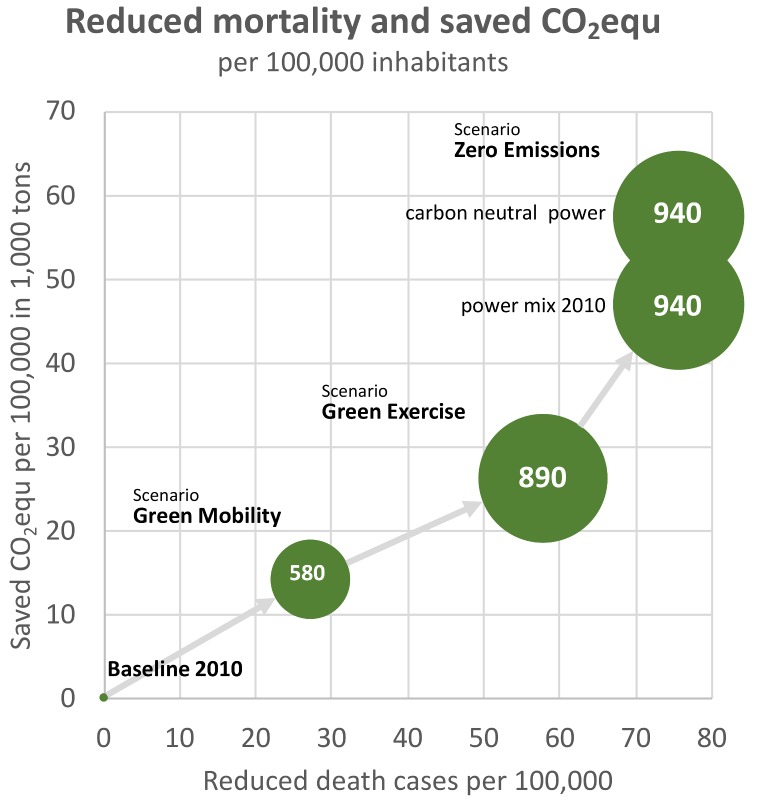
Summary of co-benefits due to CO_2_equ reduction, reduced death cases (through physical activity and improved air quality) and reduced health costs. The size of the green bubbles correspond to the numbers in white which represent the cost savings due to mortality and morbidity decreases for each scenario in 1000 €/100,000 inhabitants.

Figure 12 shows a summary of results per 100,000 inhabitants. For the Zero Emissions Scenario with a carbon neutral power mix this yields a reduction of about 60,000 t of CO_2_equ relative to the baseline, a reduction of about 75 death cases and cost savings, i.e., benefits of 940,000 € per 100,000 inhabitants and year.

## 4. Discussion

Recent literature shows positive health co-benefits from improved air quality and physical activity due to climate mitigation strategies in passenger transport (cf. [10,12]). Most of the studies end with the valuation of changes in health parameters (deaths, incidence, hospital admissions etc.) and do not include an economic assessment of these benefits in monetary terms. Some consider specific aspects like changes in air quality or cycling only to be considered [13]. There is only one study incorporating investment and operating costs of transportation policies, which are compared with benefits [21]. A reason for the lack of economic valuation could be that valuation of co-benefits needs an interplay of different disciplines and model approaches, which is a challenging task. It calls for a mutual understanding of different disciplines and models and well-considered interfaces between the models and corresponding data. As an example, some models (in our case the emission dispersion model) take many weeks to generate spatially-explicit air pollution data. Thus, only few changes in input data can cause time-consuming and costly interdependent model runs. Furthermore, a wide set of data from different sources has to be collected and processed on different levels of detail.

By conducting the underlying study, we meet these challenges despite the expected obstacles and apply a multi-model approach that considers all modes of passenger transport (cycling, walking, public transport and car travel). We consider and model commuters transport (within the city borders). Our approach regards and valuates direct investment and operating costs of mitigation policies as well as monetarized benefits from reduced health costs for private households and the public sector. Furthermore, the approach incorporates a macroeconomic feedback analysis of indirect costs and benefits due to the interrelation of different sectors within the national economy.

Jack and Kinney [70] emphasize that available estimates of co-benefits have only rarely found their way into policy considerations and suggest retrospective analysis instead of future modelling to enhance credibility of results. With this study we take an alternative route to this challenge by starting from the passenger transport targets as agreed to by city parliaments and calculate the health co-benefits of these mitigation policies. Further, we put high emphasis in enhancing the measures especially in those areas where high health benefits are to be expected to provide environmental policy makers an additional health incentive for increasing their effort.

Our results show that this effort is worthwhile because benefits from reduced death and incidence cases occur for all scenarios, as well as substantial decreases of CO_2_equ emissions and remarkable health cost savings per year. The macroeconomic analysis shows weakly negative effects for employment and GDP but positive effects on welfare. Especially when accounting for intangible dimensions, the evaluated co-benefits can compensate the potential economy-wide welfare losses from climate change impacts in Austria, which have been monetized recently in a comprehensive climate change impact assessment [71].

Still, it can be assumed that results represent an underestimation due to lack of data or appropriateness of modelling. These include psychological benefits due to more physical activity, reduced noise, increased social interaction (e.g., when switching from car to public transport) or increased green spaces (when sealed areas are rebuilt as is included in the scenarios). Such further aspects have been evaluated e.g., by Mueller et al. [1], de Nazelle et al. [72] and Boniface et al. [73]. By using qualitative methods like systematic surveys, the assessment of these costs and benefits could be amended. A further underestimation occurs because climate and congestion costs have not been included in the study. Noise costs include health costs and costs of annoyance. Applying an average cost factor per passenger-km [74] of 1.7 € per 1000 p-km would yield cost savings of 3 to 5.5 million € a year. The same procedure for external climate costs leads to cost savings that would range from 5 to 30 million € per year for the GM scenario and 10 to 55 million € per year for the GE and ZE scenario depending on the climate change scenario. Due to these uncertainties, broad ranges of cost factors not including specific spatial distribution, infrastructure conditions and exposure to the population these numbers were not included in the result tables. Further research could be done by using specific data from NEMO to calculate spatially specific changes of noise and merging these results with population data. The value of statistical life (VSL) or the value of a life year (VOLY) is mostly done by Willingness-to-Pay analysis, which is seen to be of considerably varying results (cf. Xia et al., [12]). However we included the VOLY concept in our analysis to depict the intangible costs. As we based our calculations on the YLL and YLD values which were not available for all diseases, changes in intangible costs can be seen as the lower bound. While there are increasing voices that clearly think that the VOLY concept is more meaningful in the context of air quality [57,58,75] other studies still apply the VSL approach [76,77]. Bollen et al. [77] argue that the VSL would follow the precautionary principle and would be statistically more reliable. We therefore applied two VOLY factors and contrasted them to the VSL results. Changes in morbidity and sick leaves are also considered in the Macroeconomic Model (5) depicting their implications on GDP, employment and welfare.

Another limitation of the approach presented is that we do not consider simultaneous changes in other health-related aspects. Within the broader research effort that this analysis was embedded in, health effects of changed dietary behavior (to foster greenhouse gas mitigation) have also been evaluated. Including combined health effects due to diet and mobility behavioral changes could be subject of future research.

## 5. Conclusions

Modelling co-benefits of mitigation policies in the transportation sector is a complex process requiring the interplay of different disciplines and sensible handling of data. The present study demonstrates that it is worth expanding the effort in quantifying such co-benefit effects of greenhouse gas emission mitigation policies. We do so for the three biggest Austrian urban areas. We directly transfer policy decisions (mitigation targets set by the local governments) into changes in physical activity and air quality and provide information on investment and operating costs but also on health co-benefits from reduced deaths and diseases covering reduced direct and indirect health costs) as well as on macroeconomic effects. As complex economic assessment tools had previously only been employed mainly for measures in the area of cycling (HEAT model [18]) or for changes in air quality [13], this study amends current literature by applying a multi-model comprehensive approach to calculate co-benefits from the perspectives of different economic actors, regarding all modes of passenger transport as well as domestic and commuter transport. Results show remarkable reductions of deaths in the cities, with stronger effects due to increased physical activity than to improvement of air quality. The benefits are highest for the Zero Emission Scenario and are especially due to reductions in NO_2_ when replacing the entire current vehicle fleet with electric vehicles. Benefits tend to be even higher when additional components would be included, such as changes in psychological effects from increased physical activity, green space or decreased noise exposure due to reduced motorized private transport. We also find that from a macroeconomic perspective, greenhouse gas mitigation policies in urban passenger transport clearly induce a strong positive welfare effect, when also accounting for co-benefits. These effects are remarkably higher when including the reduction of intangible costs, i.e., considering that transport policy due to its health effects is extending life expectancy which we also quantify in monetary terms by means of the value of life years (VOLY). We conclude that it is worthwhile to make the effort to assess co-benefits of climate mitigation policies in urban areas, because the numbers are significant and warrant consideration in decision making.

## Figures and Tables

**Table 1:** Example of shifting car trips (conventional drive) in Vienna for the scenario ‘Green Mobility’ to other modes of transport by different trip lengths.

	Shifted Trips [%]	Pedestrian	Bike	Public Transport	E-Car	E-Bike
*Domestic Transport*						
0.01–0.99 km	65	8%	83%	7%	1%	1%
1.00–1.99 km	50	5%	85%	8%	1%	1%
2.00–2.99 km	45	0%	75%	23%	1%	1%
3.00–4.99 km	35	0%	55%	43%	1%	1%
5.00–9.99 km	25	0%	45%	53%	1%	1%
*Commuter Transport*						
10.00–14.99 km	20		5%	93%	1%	1%
>=15 km	20			99%	1%	0%

**Table 2:** Hazard risks (HR) for the more active group in each city and scenario according to their changes in activity levels for atraumatic mortality.

City	Graz		Linz		Vienna	
Scenario	GM	GE	GM	GE	GM	GE
Hazard Risk (HR)	0.773	0.770	0.765	0.767	0.772	0.769

**Table 3:** Relative Risks (RR) and Hazard Risks (HR) (95% CI) for different health endpoints per 10 µg/m^3^ increase of NO_2_, PM_2.5_ and PM_10_.

Health endpoints	NO_2_	PM_2.5_	PM_10_
Atraumatic mortality	1.04 (1.02–1.06) [44]	1.05 (1.01–1.09) [44]	1.045 (1.029–1.060) ** [46]
Cardiovascular mortality	1.13 (1.09–1.18) [44]	1.20 (1.09–1.31) [44]	-
Respiratory mortality	1.03 (1.02–1.03) [44]	1.05 (1.01–1.09) [44]	-
Coronary events (HR) incidence (acute myocardial infarction)	-	1.13 (0.98–1.30) [45]	1.12 (1.01–1.25) [45]
Lung cancer (HR) incidence	-	1.18 * (0.96–1.46) [43]	1.22 (1.03–1.45) [43]
Cardiovascular hospital admissions (all ages)	-	-	1.013 (1.007–1.019) [41]
Respiratory hospital admissions (all ages)	-	-	1.013 (1.001–1.025) [41]

* HR per 5 µg/m^3^, ** RR from Vienneau et al. [46] based on Hoek et al. [42].

**Table 4:** Sick leaves for diseases triggered by air pollution and physical activity.

Disease	Mean Number of Days of Sick Leave per Year
Myocardial infarction	37.5
Lung cancer	75.0

Source: Leoni [64].

**Table 5:** Changes in vehicle mileage for the scenarios Green Mobility and Green Exercise (correspondingly Zero Emission scenario) relative to the baseline 2010.

Changes in mileage by city	Green Mobility	Green Exercise (Zero Emission)
Changes in car-km relative to the baseline (million km and %)		
Vienna	−1306 (−32%)	−2241 (−55%)
Graz	−276 (−24%)	−564 (−48%)
Linz	−166 (−16%)	−416 (−41%)
Changes in bus-km relative to the baseline (%)		
Vienna	17.8%	19.8%
Graz	15.9%	34.6%
Linz	4.7%	21.6%

**Table 6:** Additional physical activity due to increased walking and biking (number of persons, minutes per person and week).

Scenario	Pedestrian		Biker		E-Biker	
	Persons	Minutes	Persons	Minutes	Persons	Minutes
Graz GM	675	+167	18,599	+178	734	+173
Graz GE	8666	+217	37,673	+206	3101	+263
Linz GM	588	+187	10,872	+237	416	+187
Linz GE	3954	+227	20,398	+233	1754	+287
Vienna GM	5014	+212	153,939	+180	4702	+160
Vienna GE	71,013	+239	254,449	+214	12,662	+258

**Table 7:** Changes in mortality due to increased physical activity and changes in air quality relative to the baseline for all cities (death cases).

Death Cases	Cause (activity, air quality)	Green Mobility	Green Exercise	Zero Emissions
**Atraumatic Mortality**	Physical Activity	−417	−891	−891
	NO_2_	−88	−185	−421
	PM_2.5_	−48	−58	−65
	PM_10_	−41	−70	−91
**Cardiovascular Diseases**	NO_2_	−135	−284	−647
	PM_2.5_	−91	−110	−123
**Respiratory Diseases**	NO_2_	−4	−8	−18
	PM_2.5_	−3	−3	−4

**Table 8:** Changes in death cases, incidence numbers, hospital admissions and YLD (displayed in brackets) for the three cities and scenarios due to changed air quality.

Changes in	Graz			Linz			Vienna		
	GM	GE	ZE	GM	GE	ZE	GM	GE	ZE
Cardiovascular mortality NO_2_	−26	−48	−99	−1	−14	−50	−107	−222	−498
Respiratory mortality NO_2_	−1	−1	−3	0	−1	−2	−3	−6	−13
Myocardial infarction Incidence PM_10_	−4 (−0.07)	−8 (−0.1)	−10 (−0.1)	−3 (−0.02)	−6 (−0.05)	−7 (−0.08)	−20 (−0.3)	−34 (−0.5)	−43 (−0.7)
Lung cancer (HR) Incidence PM_2.5_	−2 (−0.2)	−3 (−0.3)	−4 (−0.4)	−1 (−0.03)	−1 (−0.09)	−1 (−0.2)	−20 (−2.0)	−23 (−2.3)	−25 (−2.5)
Cardiovascular hospital admissions PM_10_	−8	−14	−17	−3	−7	−12	−34	−57	−72
Respiratory hospital admissions PM_10_	−5	−8	−10	−2	−4	−7	−18	−31	−39

**Table 9:** Health and intangible costs relative to baseline due to changes in mortality and morbidity (all values per year).

Direct and indirect health costs and intangible costs	Green Mobility	Green Exercise	Zero Emissions
**Changes due to improved air quality**			
**Direct costs (in €1000)**			
Acute in-patient treatment including medicine	−2850	−3940	−4680
**Indirect costs**			
Morbidity (work absence in days)	−2740	−3730	−4350
Morbidity (in 1000 €)	−280	−380	−440
Mortality (number of persons)	−135	−284	−647
Mortality (in 1000 €)	−4290	−4400	−4600
**Changes due to increased physical activity**			
**Direct and indirect costs (in 1000 €)**	−4350	−9200	−9200
Mortality (number of persons)	−417	−891	−891
**Total changes of direct and indirect costs (air quality and increased physical activity) (in 1000 €)**	−11,770	−17,920	−18,920
**Changes in intangible costs due to improved air quality and increased physical activity (in 1000 €)**			
VOLY (€43,000)	−352,700	−715,600	−738,500
VOLY (€60,000)	−490,400	−995,000	−1,026,800
VSL (€1,650,000)	−910,900	−1,938,900	−2,537,400

**Table 10:** Co-benefits of climate mitigation in urban transportation for the three scenarios and cities.

Summary of co-benefits	Green Mobility	Green Exercise	Zero Emissions
**Mortality** (death cases)			
Air Quality	−135	−284	−647
Physical Activity	−417	−891	−891
**GHG emissions**			
(t CO_2_equ)	−289,680	−534,260	−956,500
**Direct and indirect health costs**			
(1000 € per year)	−11,800	−18,000	−19,000
**Intangible costs VSL**			
(1000 € per year)	−910,900	−1,938,900	−2,537,400
**Macroeconomic Effects [%]**			
GDP	−0.01%	−0.00%	−0.07%
Welfare	+0.2%	+0.3%	+0.2%
Employment	+0.1%	+0.1%	+0.1%

## Supplementary Materials

The following are available online at http://www.mdpi.com/1660-4601/15/5/880/s1, Supplementary Material for Models.

## Appendix A

**Table A1:** Sector description of CGE model.

NACE Code	Activity/Industry	Model Code
V01	Crop and animal production, hunting and related service activities	AGRI
V02	Forestry and logging	FORE
V86	Human health activities	HEAL
V87_88	Residential care activities; Social work activities without accommodation	
V36	Water collection, treatment and supply	WATE
V37_39	Sewerage; Waste collection, treatment and disposal activities; materials recovery; Remediation activities and other waste management services	WAST
V35	Electricity, gas, steam and air conditioning supply	ELEC
V19	Manufacture of coke and refined petroleum products	COKE
V28	Manufacture of machinery and equipment n.e.c.; Manufacture of electrical equipment	MACH
V29	Manufacturing of cars	MACA
V30	Manufacture of other transport equipment	MAVE
V41	Construction of buildings	BUIL
V42	Civil engineering	CIEN
V43	Specialised construction activities	CONT
V68	Real estate activities	REAL
V71	Architectural and engineering activities; technical testing and analysis	ARCH
V45	Wholesale and retail trade and repair services of motor vehicles and motorcycles	TRCA
V49	Land transport and transport via pipelines	LTRA
V50	Water transport	WTRA
V51	Air transport	ATRA
V52_53	Warehousing and support activities for transportation; Postal and courier activities	STRA
V10, V12	Manufacture of food products; Manufacture of tobacco products	FOOD
V11	Manufacture of beverages	BEVE
V16	Manufacture of wood and of products of wood and cork, except furniture; manufacture of articles of straw and plaiting materials	WOOD
V17	Manufacture of paper and paper products	PAPE
V20	Manufacture of chemicals and chemical products	CHEM
V21	Manufacture of basic pharmaceutical products and pharmaceutical preparations	PHAR
V22_23	Manufacture of rubber and plastic products; Manufacture of other non-metallic mineral products	PLAS
V24	Manufacture of basic metals	META
V25	Manufacture of fabricated metal products, except machinery and equipment	MAME
V27	Manufacture of electrical equipment;	MAEL
V13_14, V18, V26, V31_33	Rest of manufacturing (Manufacture of textiles; Manufacture of wearing apparel; Printing and reproduction of recorded media; Manufacture of computer, electronic and optical products; Manufacture of furniture; Other manufacturing; Repair and installation of machinery and equipment)	RMAN
V15	Manufacture of leather and related products	LEAT
V46_47	Wholesale trade, except of motor vehicles and motorcycles; Retail trade, except of motor vehicles and motorcycles	TRAD
V64	Financial service activities, except insurance and pension funding	FINA
V65	Insurance, reinsurance and pension funding, except compulsory social security	INSU
V66	Activities auxiliary to financial services and insurance activities	AFIN
V84	Public administration and defence; compulsory social security	PUBL
V55_56	Accommodation; Food and beverage service activities	ACCO
V79	Travel agency, tour operator and other reservation service and related activities	TRAV
V90	Creative, arts and entertainment activities	ENTE
V91	Libraries, archives, museums and other cultural activities	CULT
V93	Sports activities and amusement and recreation activities	SPOR
V03, V05_09	Fishing and aquaculture; Mining of coal and lignite, Extraction of crude petroleum and natural gas, Mining of metal ores, Other mining and quarrying, Mining support service activities	REXT
V58	Publishing activities	RECR
V59_60	Motion picture, video and television programme production, sound recording and music publishing activities; Programming and broadcasting activities	
V92	Gambling and betting activities	
V69_70	Legal and accounting activities; Activities of head offices, management consultancy activities	SCIE
V72	Scientific research and development	
V74_75	Other professional, scientific and technical activities; Veterinary activities	
V61	Telecommunications	TELE
V62_63	Computer programming, consultancy and related activities; Information service activities	
V77	Rental and leasing activities	RSER
V78	Employment activities	
V80_82	Security and investigation activities; Services to buildings and landscape activities; Office administrative, office support and other business support activities	
V85	Education	
V94	Activities of membership organisations	
V96	Other personal service activities	
V97_98	Activities of households as employers of domestic personnel; Undifferentiated goods- and services-producing activities of private households for own use	
V99	Activities of extraterritorial organisations and bodies	
V73	Advertising and market research	ADVE
V95	Repair of computers and personal and household goods	REPA
