# Supplementary material for: Evaluating Health Co-Benefits of Climate Change Mitigation in Urban Mobility
Source: Int J Environ Res Public Health. 2018 Apr 28;15(5):880. doi: 10.3390/ijerph15050880 (PMC5981919; doi:10.3390/ijerph15050880)
Supplement: Supplementary file 1 [file ijerph-15-00880-s001.pdf]

International Journal of
Environmental Research and Public Health

MDPI

Supplementary Material

Evaluating Health Co-Benefits of Climate Change Mitigation in Urban Mobility

Brigitte Wolkinger, Willi Haas, Gabriel Bachner, Ulli Weisz, Karl Steininger, Hans-Peter Hutter, Jennifer Delcour, Robert Griebler, Bernhard Mittelbach, Philipp Maier and Raphael Reifeltshammer

S1: GRAL dispersion model

GRAL is a 3D Lagrangian particle model developed at the Institute for Internal Combustion Engines and Thermodynamics of Graz University of Technology, Austria [1–3]. Typical applications range from the calculation of pollutant dispersion from single sources in micro-scale domains (below 1 km x 1 km) to the calculation of pollutant dispersion for a multitude of sources at meso-scale domains (up to several tens of kilometers across).

Lagrangian models, such as GRAL, provide an alternative method (with respect to Eulerian ones) for simulating atmospheric diffusion. They are called Lagrangian because the modelling is performed by following fluid elements during the flow instead of solving transport equations over a fixed grid. In particular, Lagrangian particle models simulate the pollutant behaviour by generating a set of dynamic atmospheric trajectories of pollutant mass. In other words, a certain number of fictitious particles like 'tracers' is employed, i.e., particles are released inside the computational domain and their motion is tracked by solving appropriate equations describing their trajectories. Then, the concentration in the desired Eulerian fixed grid can be computed as the density of the trajectory points. In particular, in each cell and for each time step, the instantaneous concentration can be obtained by summing up the masses of the particles being located in the same cell at that time and dividing that number by the volume of the cell.

In order to compute particle trajectories, GRAL needs to know the velocity components over the entire computational domain. To get the velocity components, GRAL can optionally use flow fields computed by the prognostic wind field model GRAMM, by interpolating the output of the latter over its own grid, or can compute wind velocities by itself with a simplified flow solver. In this case and to obtain all the parameters related to turbulent phenomena in general, the code utilizes classified meteorological situations (wind speed classes, wind direction, stability classes such as Pasquill-Guifford-Turner or OENORM [4–8]), while a meteorological pre-processor calculates the friction velocities, the Monin-Obukhov length, the standard deviations of the normal components of the wind speed and the dissipation rate of the turbulent kinetic energy. The pre-processor is based mainly on the work of Golder [9], Venkatram [6] , Zannetti [10]. Details on the implementation within the GRAL modelling chain can be found in Oettl [11].

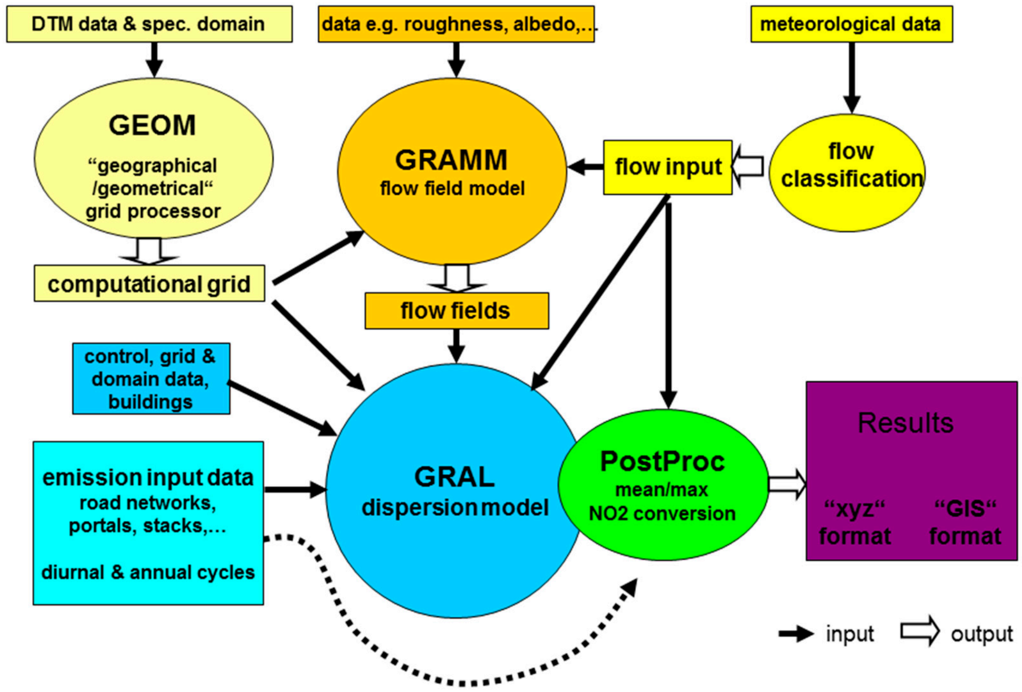

Figure S1. GRAMM/GRAL system processing chain and data flow.

GRAL can simultaneously calculate line sources (3D, also bridges), area sources, point sources, and tunnel portals. The model system uses special algorithms to account for low wind or calm conditions. Transport and diffusion of PM_{10} and NO_x are treated as chemically inert. The formation of secondary organic and inorganic PM is not considered.

S2: NEMO (Network Emission Model)

The Network Emission Model (NEMO) has been developed for the calculation of traffic related emissions in road networks. It combines a detailed calculation of fleet composition and emission simulation. Typical applications are computations for emission inventories in urban and regional applications, the evaluation of measures such as environmental zones, or the evaluation of complex infrastructure projects.

Based on its flexible model structure, NEMO is applicable for the evaluation of different scenarios. The features implemented into the model enable for example the representation of effects that influence driving behaviour (e.g., traffic calming) or of special actions having an effect on the fleet composition (e.g., promotion programs for diesel vehicles with exhaust aftertreatment) on the fleet emissions.

The model physics is based on the Passenger car and Heavy duty vehicle Emission Model (PHEM); a detailed simulation tool for energy consumption and emissions of passenger cars and heavy duty vehicles [12–16]), global modelling of scenarios concerning emission and fuel consumption in the transport sector (GLOBEMI, [17]) and the Handbook emission factors for road transport (HBEFA3.1, 2010 [18]). PHEM has been developed in several international and national projects, namely the EU 5th research framework program ARTEMIS, the COST 346 initiative and the German-Austrian-Swiss cooperation on the Handbook of Emission Factors [18].

NEMO is able to compute emissions of NO_x, PM_{10}, (exhaust and non-exhaust), CO_2, CO, NO_2, SO_2, NMHC, benzene and others for each road section. Two PM_{10} non-exhaust emission factor sets can be used optionally, either based on Gehrig et al. [19] or Düring et al.[20]. In practice, roads are divided into sections (often up to thousands) to account for their exact position (horizontally and vertically) and road gradient. Further model input are traffic volumes, fleet composition for the

specific years, and average vehicle speed. If the exact fleet composition is unknown, NEMO uses a fleet composition based on registration statistics [21] and the type of the road.

The basic physical principle of the NEMO approach for the simulation of the vehicle emissions is the strong correlation of the engine specific emission behavior (emissions in grams per kilowatt-hour engine work) with the cycle average engine power in a normalized format, which is valid for all engines inside a certain vehicle category, engine concept and emission standard (Figure S2).

NEMO consists of three major modules (Figure S2):

- The Fleet Module calculates the detailed fleet composition concerning different vehicle types and engine concepts (e.g., gasoline or diesel), size-class (differentiating factor: capacity or maximum allowed gross weight), engine sizes, emission standards (e.g., Euro 2) and exhaust aftertreatment. This information is based on the Austrian registration statistics and drop out probabilities for the respective year of interest.
- The Emission Module simulates the vehicle emissions for the different layers in the vehicle fleet based on the respective vehicle specifications, driving cycle parameters and engine specific emission functions. These functions are based on post-processed simulation results of the model PHEM and data from HBEFA3.1. The simulation results of PHEM are based on engine test bed and chassis dynamometer emission measurements at the Institute for Internal Combustion Engines and Thermodynamics (IVT) at TU Graz.
- The Road Network Module combines the information of the Fleet and Emission modules and road network data to yield finally the emission output on the road section under consideration.

Further detailed information can be found in Rexeis and Hausberger [22,23].

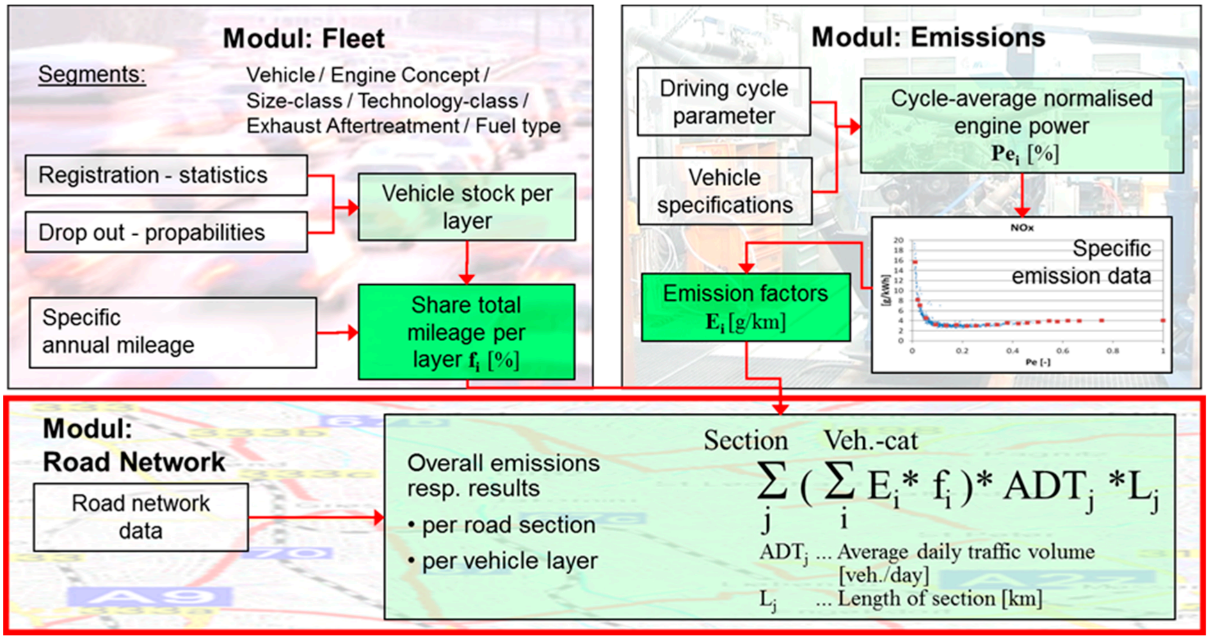

Figure S2. Schematic of the Network Emission Model—NEMO.

S3: Detailed concentration level maps

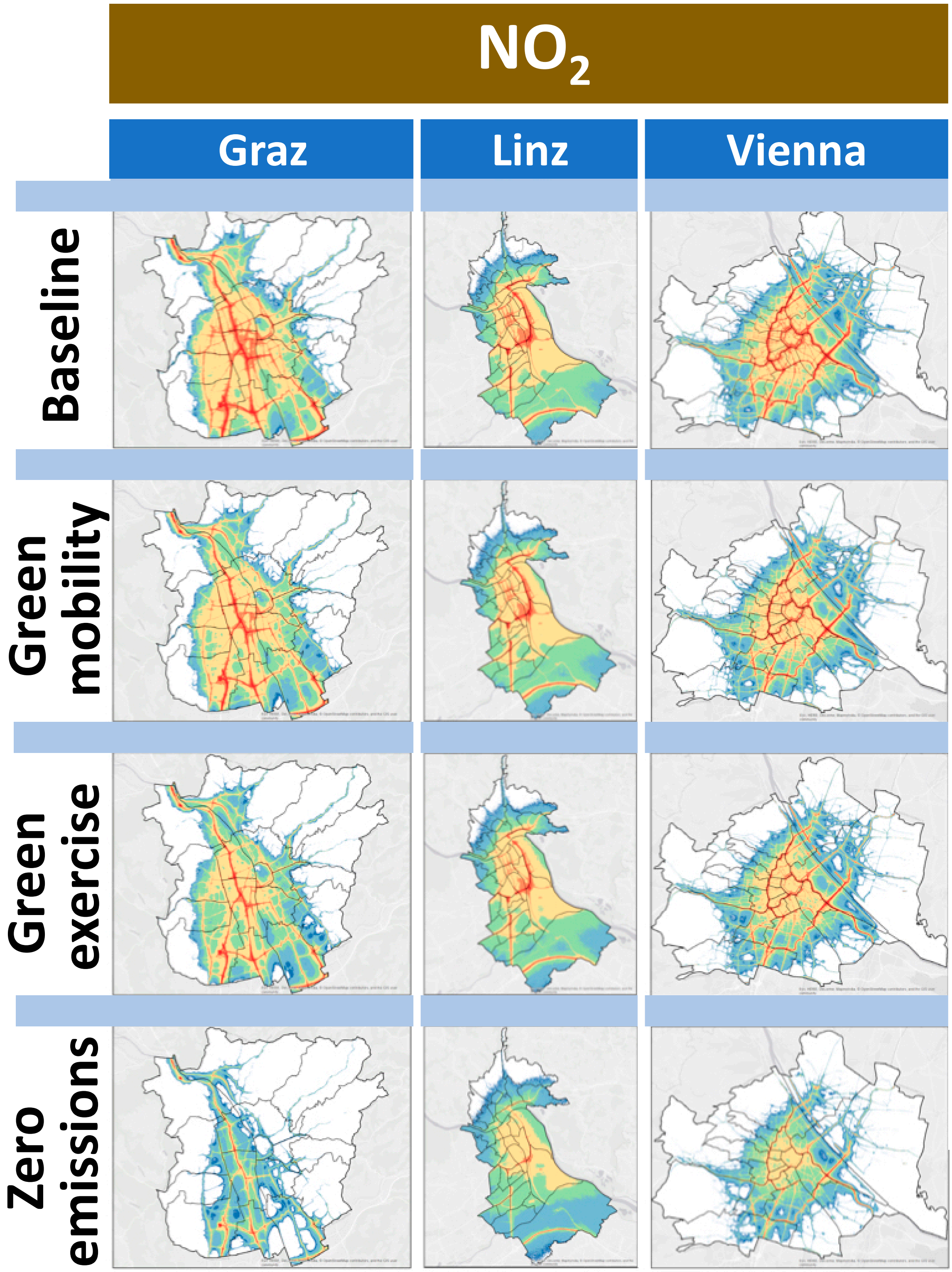

Figure S3. Concentration level maps for NO_2 for all scenarios and the baseline.

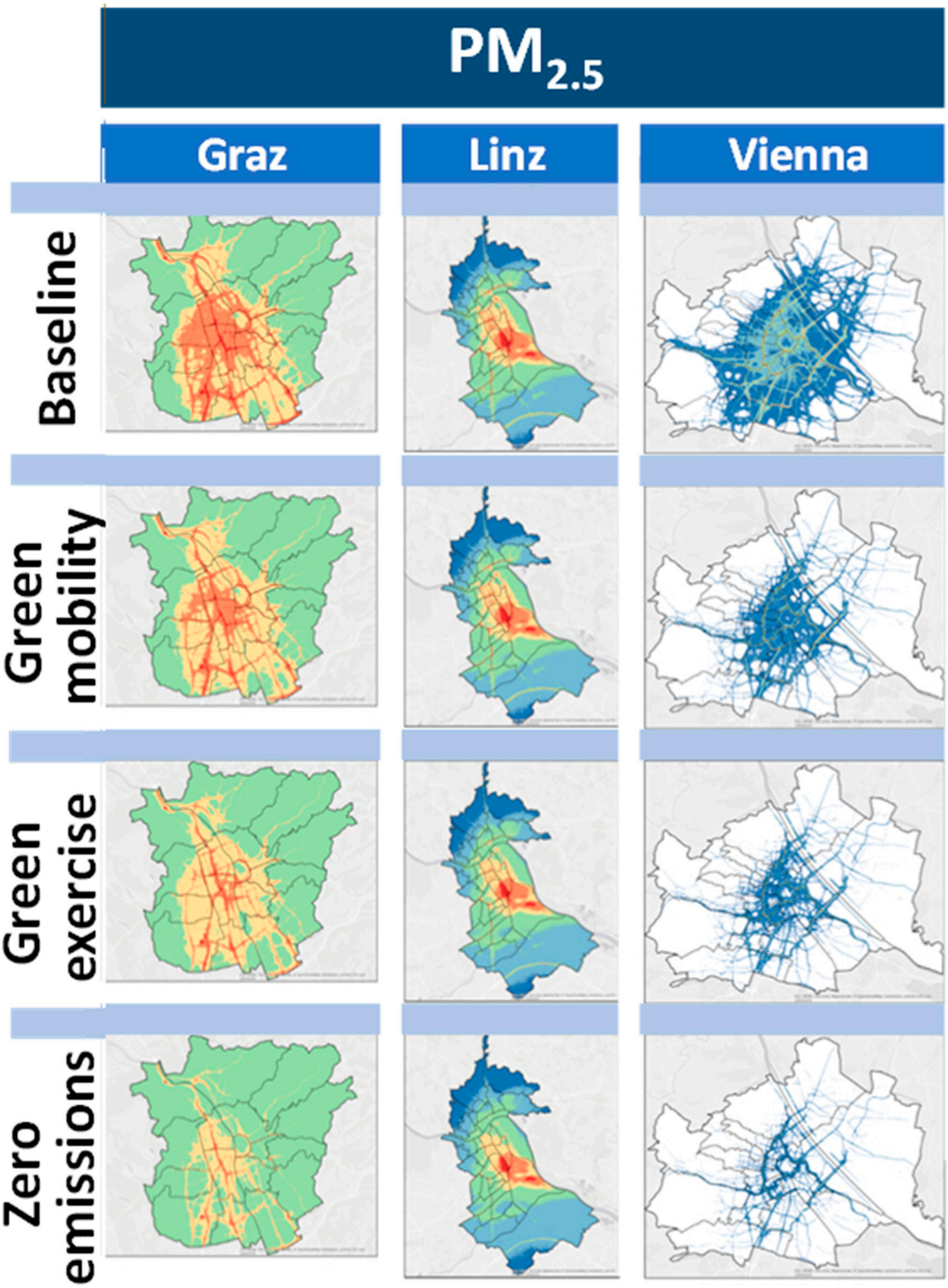

Figure S4. Concentration level maps for $PM_{2.5}$ for all scenarios and the baseline.

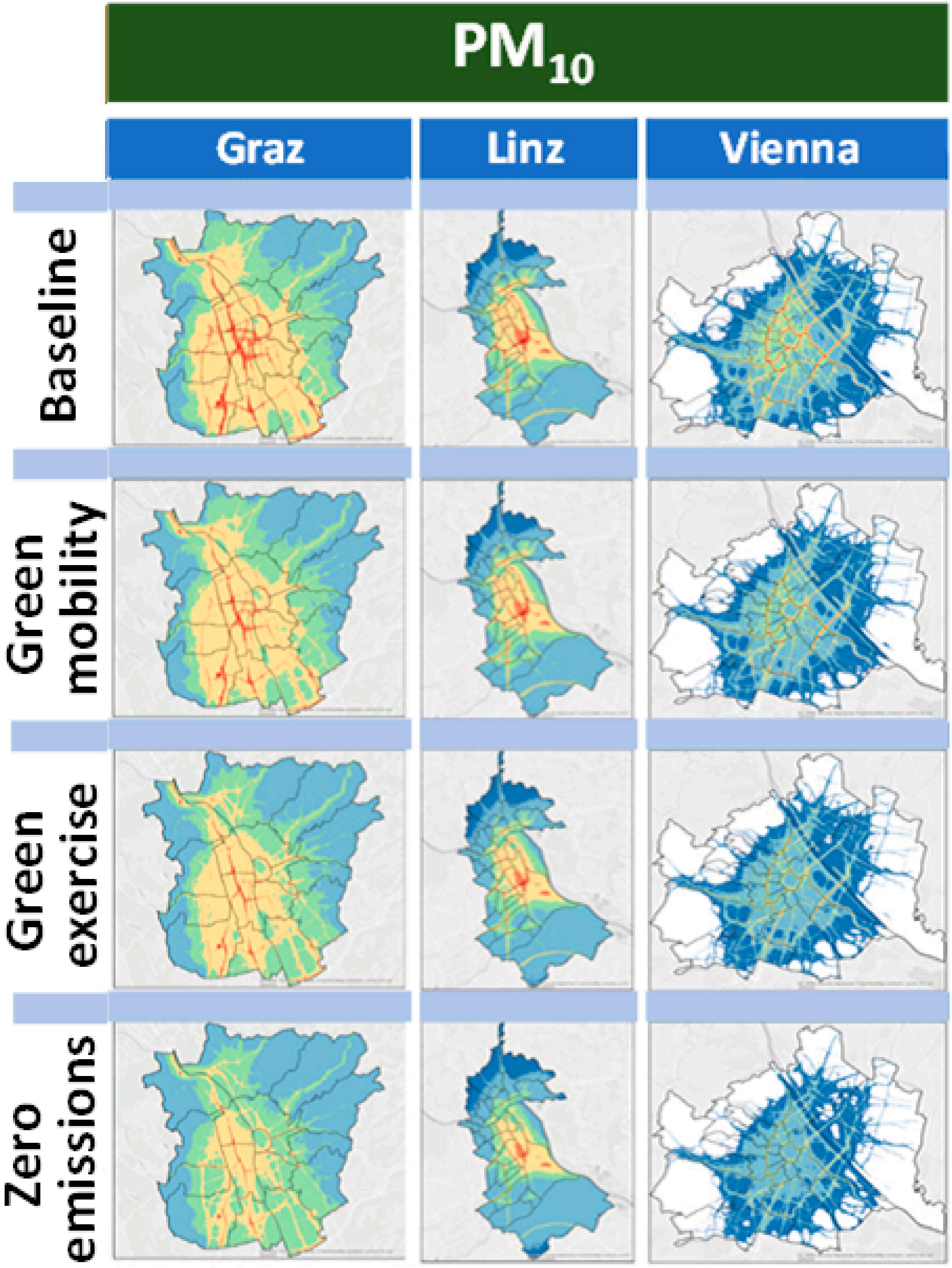

Figure S5. Concentration level maps for PM_{10} for all scenarios and the baseline.

S4: Transport Modelling Tool

S4.1. Baseline

The Transport Modelling Tool is based on a bottom up approach starting from the mobility surveys conducted for the three Austrian cities Graz, Linz and Vienna [24–27]. These surveys yield mobility data for the residential population on working days, for trips within the cities and commuter trips out of the city and include the following parameters:

- Number of trips by category of trip length and mode (#)
- Modal Share (%)
- Number of trips per person and day (#/d/p)
- Average trip length by transport mode (km)
- Average trip duration by transport mode (h)

To be able to consider the total passenger transport within the city borders commuter trips from outside into the cities are appraised using the Commuter Statistics for Austria [28]. As trips on weekends are not included in the surveys a factor of 87% for trips on Saturdays and 65% on Sundays were applied from the literature [29]. In the next step these daily trips were upscaled to yield the transport for the baseline year by multiplying trips by 332 which is a common factor considering the different number of trips on working days and weekends.

Transport performance (p-km) and mileage (vehicle-km) were calculated by multiplying the number of trips by average trip lengths by mode and distance category for the baseline 2010 for each city.

S4.2. Modelling the transport effects for the scenarios

The scenarios in the underlying study are based on the modal share targets for 2020/2025 the local and regional governments have decided on. These overall target shares by transport mode can be reached by shifting trips from one mode (motorized individual transport) to other modes (public transport, bicycle or pedestrian) within a certain distance category. This has been done by expert appraisement, which first determines the share of car trips within a distance category that can be shifted to other modes of transport and second decides which other modes of transport the shifted trips are distributed to. For example for Vienna for the Green Exercise (GE) scenario 90% of car trips within the shortest distance category are shifted to other modes of transport. Thereof 60% of trips are travelled by foot, 38% by bike and 1% each by e-car and e-bike. The share of shifted car trips and the distribution to other modes of transport for the GE scenario for each city are shown in Table S1 to Table S3.

Table S1. Shifted car trips (conventional drive) to other modes of transport by different trip lengths (GE-Vienna).

Trip Length	Shifted trips [%]	Pedestrian	Bike	Public transport	e-Car	e-Bike
Domestic Transport (km)						
0.01–0.99	90	60%	38%	0%	1%	1%
1.00–1.99	85	40%	58%	0%	1%	1%
2.00–2.99	75	10%	88%	0%	1%	1%
3.00–4.99	65	0%	80%	18%	1%	1%
5.00–9.99	50	0%	48%	50%	1%	1%
Commuter Transport (km)						
10.00–14.99	20		5%	88%	1%	6%
≥ 15	20		0%	89%	1%	10%

Table S2. Shifted car trips (conventional drive) to other modes of transport by different trip lengths (GE-Graz).

Trip Length	Shifted trips [%]	Pedestrian	Bike	Public transport	e-Car	e-Bike
Domestic Transport (km)						
0.01–0.99km	90	60%	38%	0%	1%	1%
1.00–1.99km	80	40%	58%	0%	1%	1%
2.00–2.99km	60	10%	88%	0%	1%	1%
3.00–4.99km	50	0%	60%	38%	1%	1%
5.00–9.99km	40	0%	35%	63%	1%	1%
Commuter Transport (km)						
10.00–14.99	20		5%	88%	1%	6%
≥ 15	20		0%	89%	1%	10%

Table S3. Shifted car trips (conventional drive) to other modes of transport by different trip lengths (GE-Linz).

Trip Length	shifted trips [%]	pedes-trian	bike	public transport	e-car	e-bike
Domestic Transport						
0.01–0.99	90	60%	38%	0%	1%	1%
1.00–1.99	75	30%	68%	0%	1%	1%
2.00–2.99	60	10%	88%	0%	1%	1%
3.00–4.99	50	0%	60%	38%	1%	1%
5.00–9.99	30	0%	35%	63%	1%	1%
Commuter Transport						
10.00–14.99	15		5%	88%	1%	6%
≥ 15km	15		0%	89%	1%	10%

These changes for each scenario yield the new number of trips by distance category and correspondingly changes in overall transport performance and mileage applying the same procedure for upscaling like for the baseline. Additionally the transport plans of the local and regional parliaments contain target levels for the occupation rate for local and commuter transport which are applied within the Transport Modelling Tool. The target occupation rates of the mobility plans are given in Table S4 for the Green Mobility (GM), Green Exercise (GE) and Zero Emission (ZE) scenario.

Table S4. Targets for car occupation rates for all cities.

City	Baseline	GM	GE	ZE
Vienna-domestic transport	1.29	1.50	1.50	1.50
Vienna-commuters	1.25	1.35	1.50	1.50
Graz-domestic transport	1.27	1.50	1.50	1.50
Graz-commuters	1.20	1.35	1.50	1.50
Linz-domestic transport	1.30	1.50	1.50	1.50
Linz-commuters	1.27	1.35	1.50	1.50

Shifts from car transport to other modes of transport as well as changes in the occupation rate of remaining car trips lead to changes in overall mileage (vehicle-km) while the transport performance (passenger-km) remains constant as it is the underlying assumption for all scenarios.

Thus the transport service as numbers of daily trips travelled by one person is not changed. Table S5 exemplifies a result table for the GE scenario for Vienna.

Table S5. Example for a result table for Scenario GE-Vienna (Difference to baseline).

Mileage and Transport performance	Pedestrian (p-km)	Bike (p-km)	Public transport (p-km)	Car (vehicle-km)	Fellow passenger car (p-km)	e-Vehicles (p-km)
1000 km per working day	266	2,135	1,554	−3,670	−420	135
1000 km per year (working days)	65,148	522,962	380,741	−899,087	−102,785	33,022
1000 km per year (weekends included)	23,816	191,181	139,188	−328,681	−37,575	12,072
total 1000km (residential population)	88,964	714,143	519,930	−1,227,769	−140,361	45,094
1000 km (commuters from outside)	-	69,940	669,429	−1,013,142	13,994	259,779
1000 km (overall)	88,964	784,083	1,189,359	−2,240,911	−126,367	304,872

S4.3. Calculating Changes in energy demand and GHG

Results of changed transport performance and mileage are then used to calculate changes in energy consumption and greenhouse gas emissions (GHG). Therefore changes in energy demand are calculated using energy and emission coefficients displayed in vehicle-km for car and bus based on Hausberger [30] as displayed in Table S6.

Table S6. Energy and emission coefficients by mode of transport

Transport Mode	Energy coefficients (kWh/p-km) or (kWh/v-km)	GHG coefficients (kg CO_2equ/kWh)
Public transport (p-km)		
Bus	0.12	0.266
Train	0.09	0.210
Electric	0.06	0.210
Motorized Individual transport (v-km)		
Electric vehicles	0.21	0.210
Car (diesel/petrol)	0.65	0.270

S4.4. Calculating Changes in Physical Activity

Besides changes of transport performance and mileage as well as energy and GHG emission changes a crucial output of the Transport Modelling Tool are changes in physical activity that enter into the Health Model. Calculations start from the changes in trips for pedestrian and bike with the conservative underlying assumption that each person shifts only one trip (there and back). Based on average velocities for the transport modes and average trip length per distance category additional time for physical activity is calculated (Δ min/person/week). The applied average velocities are 6 km/h for pedestrian, 15 km/h for bike and 22 km/h for e-bike.

S5: Economic Assessment Tool

The Economic Assessment Tool valuates costs and benefits that occur due to changes in mobility and correspondingly changes in physical activity and air pollution. It mainly assesses internal private net-benefits (benefits-costs) and public net-benefits for investment and operation of climate mitigation measures and savings of private expenditures due to decreased car trips.

S5.1. Changes in investment and corresponding operating costs

A detailed evaluation of investment and operating costs for each city-specific measure (e.g. specific shared space project) would have gone beyond the scope of the research project. Therefore we draw on former studies of evaluating transport measures for Austria which provide specific cost factors per additional passenger-km for different public transport modes and information on required investment to obtain the target changes in transport performance or mileage. The average operating costs for an increase in public transport performance due to a shift from car trips to public transport is displayed in Table S7.

Table S7. Average operating costs for additional passenger-km by public transport modes

Transport Mode	€/pkm
Train	0.07
Electric (mix tram, e-bus, underground)	
Graz	0.10
Linz	0.10
Vienna	0.15
Bus (conventional drives)	0.20
Train	0.07

The approach for calculating investment and operating costs is done for specific bundles of measures and is exemplarily explained for the increase in pedestrian, bike and e-bike traffic. The calculations start from the changes in passenger-km for each scenario and city obtained from the Transport Calculation Tool. Applying average transport performance per transport mode and year yields the number of bikes, e-bikes and pedestrians. In principle calculations are based on the new pedestrians and bikers and operating and investment costs that occur for them per year. For investments average amortization rates are applied which range between 10 and 15 years. For investments of pedestrians and bikers we consider that about 60% of additional users already own a bike. Thereof 23% will let maintain and repair their bikes. 25% of bikers will buy a new bike. Investment costs for new bikes are about 450 € and for e-bikes 2,000 €. Service costs are accounted for 72€/1,000 km. In addition, investment costs for road infrastructure e.g. for safe bike parking spaces are included, as well as effort on new shoes when people increase their pedestrian trips substantially. Table S8 depicts the sum of operating and investment costs per economic sector for the specific bundle of measures as it enters into the macroeconomic CGE model.

Table S8. Operating and investment costs per OENACE sector for increase of trips for bike, pedestrian and e-bike (M €/year)

OENACE sector		GM	GE	ZE
Operating costs (private/public) total		34.43	169.58	169.58
15	Manufacture of footwear	0.45	6.90	6.90
35	Electricity, gas, steam and air conditioning supply	0.04	1.53	1.53
42	Civil Engineering	0.07	0.23	0.23
65	Insurance	1.19	50.99	50.99
81	Services to buildings and landscape activities	0.04	0.13	0.13
95	Repair of computers and personal and household goods	32.65	109.81	109.81

Investment costs (private/public) total		10.01	81.91	81.91
27	Manufacture of electrical equipment	0.00	0.21	0.21
30	Manufacture of railway locomotives and rolling stock	6.80	71.20	71.20
42	Civil Engineering	2.12	7.99	7.99
43	Electrical, plumbing and other construction installation activities	0.00	0.10	0.10
95	Repair of computers and personal and household goods	1.08	2.41	2.41

S5.2. Changes in household expenditures

Besides, changes in private expenditures (as a part of operating costs) due to a shift from motorized individual transport to pedestrian or bike are calculated based on the Austrian Household Budget Survey 2009/2010 [31]. Household expenditures are combined with data on transport performance by mode [30] in order to calculate cost factors per saved car-kilometer or passenger-km. The cost factors applied to changes in vehicle-km or passenger-km, respectively, travelled by car and public transport are given in Table S9.

Table S9. Household Expenditures by OENACE sector per passenger-km (public transport) and vehicle-km.

Cost category	OENACE 2008 sector	€/km
Vehicle costs	45	0.12
Fuel costs	19	0.07
Maintenance	45	0.06
Other services	45	0.03
Insurance	65	0.04
Public transport	49	0.11

S6: Health Model

S6.1. Calculation of risk reduction for physical activity changes

a) Identification of relative risks based on meta-studies

First, we conducted a systematic review taking into account already existing meta-analysis. Data on the number of persons and duration of additional physical activity were obtained from the Transport Model for walking, cycling and e-cycling for the two scenarios and the cities of Graz, Linz and Vienna (see below in b).

It was assumed that there are no distinctions between the groups of people who walk the respective additional routes on foot or by bicycle or e-bike. The metabolic activity supposed was 4 MET for walking and electric cycling and 6.8 MET for cycling. The respective times per week were used to calculate MET-h / week. Based on the reviews [32–34], the hazard ratio (HR) for total mortality was calculated as a function of physical activity. The baseline activity was 7.5 METh / week (HR = 1). The baseline assumption of 7.5 MET-h / week is in line with the WHO recommendation for minimum physical activity. From the data on the relationship between physical activity in MET-h / week and the risk of total mortality (mainly due to cardiovascular disease), a consistent functional relationship was derived from the reviews cited using the following procedure: The hazard ratios and / or odds ratios (OR) were transformed and related to the respective base rate. This was necessary because both the baseline metabolic rate of the reference population and their mortality (total mortality) varied. As some results in the selected meta-analyzes were given as ORs for a certain observation period and some results as HR related to 1 year, a standardization was made for all results - as HR related to 1 year.

In a next step, the standardized mortality rates thus determined were analyzed in terms of their functional dependence on MET-h / week. There was a clear dependence on the MET-h / week, which - with an explained variance of more than 98% - served as basis for the calculation of the HR values for the respective additional physical activities by walking, e-cycling or cycling. To determine the average HR for the total number of people in the cities and scenarios that have additional physical activity, either as longer walkways, more cycling or e-cycling, the HR for each of these subgroups was determined separately and then the number of persons weighted average calculated (more accurate as a weighted geometric mean). HR was calculated for 15 movement groups (3 types of movement and 5 distance classes) per scenario and city. So a total of 90 individual calculations were performed.

b) Affected population and increased physical activity

Based on the shifts from passive mobility (car use and public transport) to increased active mobility we calculated the additional persons and their additional minutes in active mobility (see Table 6 in the main text).

c) Mortality and YLL in the three cities

Atraumatic mortality (ICD-10: A00-R99)was calculated based on the National Causes of Death Statistics [35] for each city. From the data base (http://vizhub.healthdata.org/gbd-compare/) we obtained values for the number of YLL caused by atraumatic mortality for Austria. In a next step we calculated a YLL per death ratio which was applied for the three target cities to calculate the amount of YLL per city. We assumed same ratios for all three cities as for Austria and calculated the absolute values for them based on the population.

d) Reduced Mortality, YLL and YLD in the three cities and per 100.000

We calculated the mortality risk of the entire populations of the three cities and reduced the mortality risk according to the RR for the sub-group who become active according to the scenario and the city. Thus, we could calculate reduced values for the number of death and YLL for the atraumatic mortality for the three cities and further, as a more generalized result, the results per 100.000 inhabitants for urban inhabitants.

S6.2. Calculation of risk reduction for changes in air pollution

a) Identification of relative risks based on meta-studies

Health endpoints were chosen based on available concentration response functions in the literature on long-term effects of particulate matter (PM_{10} and $PM_{2.5}$) and nitrogen dioxide (NO_2). PM_{10}, PM2.5 and NO_2 have proven to be the best effect estimates of the health effects of outdoor air pollution, with PM_{10} and NO_2 being the most prevalent in Europe. To identify effect estimates for these pollutants, we first performed a systematic review and included the existing meta-analyses.

The aim was to select reliable exposure-response relationships for the general population. In particular, meta-analyses of major international epidemiological studies published in high-ranking journals were used. The risk estimates are also based partly on Austrian data [36–38]. Table 3 in the main text describes exposure–response relationships used for the estimation of mortality and morbidity due to the selected air pollutants (per 10 $\mu g/m^3$ and 5 $\mu g/m^3$ increase).

By way of illustration: In Table 4 in the main text the end point (e.g. the cardiovascular mortality) is indicated as well as the relative magnitude of the increase by 10 $\mu g/m^3$ of the particles or of the pollutant NO_2 (exception: $PM_{2.5}$ end point lung CA: 5 $\mu g/m^3$). In addition, 95% confidence intervals for this relative risk are indicated.

Table S10. Affected population benefitting from reduced concentration levels for $PM_{2.5}$ and Graz

**Graz**	**Affected Population**	**Reduced annual mean concentration levels of $PM_{2.5}$ [μg/m³]**				
**District**	**Total number of persons**	**< −1**	**−1 to −0.5**	**−0.5 to −0.25**	**−0.25 to −0.1**	**-0.1 to 0.1**
I	3822	583	1901	1280	59	0
II	15208	709	9335	5132	32	0
III	24258	1233	9352	9209	4464	0
IV	27773	2843	24172	758	0	0
V	24868	5459	15376	4032	0	0
VI	30911	2566	23381	4964	0	0
VII	12197	631	3759	7794	12	0
VIII	13324	64	987	5131	5835	1306
IX	11058	21	275	2219	6814	1728
X	5549	0	13	189	1793	3553
XI	8977	3	117	589	3399	4869
XII	16958	63	1246	4118	6719	4813
XIII	9593	231	1481	1968	2078	3835
XIV	17625	1721	6578	2556	4740	2030
XV	13093	708	6658	1631	2634	1461
XVI	13054	828	4837	2529	2047	2814
XVII	6884	217	2122	3833	712	0
Total	255152	17880	111590	57934	41339	26409
Affected population (%)		7.01%	43.73%	22.71%	16.20%	10.35%

b) Affected population and decreased air pollution

The affected number of people who experience a specific change in exposure to specific pollutants was calculated by matching population data on the district level with the changes in the annual mean of specific pollutants (see Figure S3 in the supplementary file for a spatial distribution of concentration levels and Table S10 for the specific application).

c) Mortality, YLL and YLD in the three cities

We retrieved values for the number of YLL for atraumatic mortality and YLD from the data base (http://vizhub.healthdata.org/gbd-compare/) for cardio-vascular disease, respiratory disease and lung cancer for Austria and supplemented it with the National Cause of Death Statistics [35] and Austrian Cancer Registry [39]. Based on both data sources we calculated the mortality rate, and where available, the incidence rate. Additionally we gathered data on hospital admission [40].

Atraumatic mortality (ICD-10: A00-R99), cardio-vascular mortality (ICD-10: I00-I99) and respiratory mortality (ICD-10: J00-J99) were calculated based on the National Causes of Death Statistics [35]. The myocardial infarction incidence (ICD-10: I21-I22) was estimated by combining data from the National Causes of Death Statistics [35] and from the Austrian Hospital Admission Statistics [40]. The lung cancer incidence (ICD-10: C33-C34) was retrieved from the Austrian Cancer Registry [39]. As for physical activity data on Years Life Lost (YLL) and Years lived with Disability (YLD) were obtained from the Institute for Health Metrics and Evaluation database [41] for all above mentioned outcomes.

d) Reduced Mortality, YLL and YLD in the three cities and per 100.000

Further, we adjusted the relative risk to obtain an overall reduction rate for each of the cities, pollutants and scenarios that considers the number of population benefiting from different degrees of a reduced exposure to air pollutants (see Table S5). With this overall reduction rates we calculated all required changes for further calculations: mortality, incidences, YLL and YLD. These calculations were executed for NO_2, $PM_{2.5}$ and PM_{10}.

References

1. Oettl, D.; Sturm, P.-J.; Pretterhofer, G.; Bacher, M.; Rodler, J.; Almbauer, R. Lagrangian dispersion modeling of vehicular emissions from a highway in complex terrain. *J. AirWaste Manag. Assoc.* **2003**, *53*, 1233–1240.
2. Oettl, D.; Almbauer, R.; Sturm, P.-J.; Pretterhofer, G. Dispersion modelling of air pollution caused by road traffic using a Markov Chain - Monte Carlo model. *Stoch. Environ. Res. Risk Assess.* **2003**, 58–75.
3. Oettl, D.; Uhrner, U. Development and evaluation of GRAL-C dispersion model, a hybrid EulerianeLagrangian approach capturing NO-NO2-O3 chemistry. *Atmos. Environ.* **2011**, 839–847.
4. Pasquill, F. The estimation of the dispersion of windborne material. *Meteorol.Mag* **1961**, *90*, 33–49.
5. Turner, D. B. A diffusion model for an urban area. *J. Appl. Meteorol.* **1964**, *3*, 83–91.
6. Venkatram, A. An examination of the Pasquill-Gifford-Turner dispersion scheme. *Atmos. Environ.* **1996**, *30*, 1283–1290.
7. EPA *Meteorological Monitoring Guidance for Regulatory Modeling Applications*; 2000;
8. OeNorm *Ausbreitung von luftverunreinigenden Stoffen in der Atmosphäre - Berechnung von Immissionskonzentrationen und Ermittlung von Schornsteinhöhen*; 1996;
9. Golder, D. Relations among stability parameters in the surface layer. *Bound.-Layer Meteorol.* **1972**, *3*, 47–58.
10. Zannetti, P. *Air pollution modeling: theories, computational methods, and available software*; Springer: New York, NY, USA, 1990; ISBN 0-442-30805-1.
11. Oettl, D. *Documentation of the Lagrangian Particle Model GRAL , Graz Lagrangian Model Vs. 13.3*; Amt der Steiermärkischen Landesregierung, FA17C, Technische Umweltkontrolle: Graz, Austria, 2013;
12. Hausberger, S.; Engler, D.; Ivanisin, M.; Rexeis, M. *Emission Functions for Heavy Duty Vehicles - Update of the Emission Functions for Heavy Duty Vehicles in the Handbook Emission Factors for Road Traffic*; 2003;
13. HAUSBERGER, S.; REXEIS, M.; RODLER, J.; STURM, P. Aktuelle Emissionsfaktoren für schwere Nutzfahrzeuge. *ÖIAZ* **2003**, *148*, 114–119.
14. Hausberger, S. Simulation of Real World Vehicle Exhaust Emissions. *VKM-THD Mitteilungen* **2003**.
15. Hausberger, S.; Rodler, J.; Sturm, P.-J.; Rexeis, M. Emission factors for heavy-duty vehicles and validation by tunnel measurements. *Atmos. Environ.* **2003**, 5237–5245.
16. Hausberger, S.; Rexeis, M. Emission behavior of modern heavy duty vehicles in real world driving. *Int. J. Environ. Pollut.* **2004**, *22*, 275–286.
17. Hausberger, S. Globale Modellbildung für Emissions- und Verbrauchsszenarien im Verkehrssektor. PhD Thesis, 1997.
18. Hausberger, S.; Rexeis, M.; Zallinger, M.; Luz, R. *Handbuch Emissionsfaktoren des Straßenverkehrs Version 3.1*; TU Graz: Graz, 2009;
19. Gehrig, R.; Hill, M.; Buchmann, B.; Imhof, D.; Weingartner, E.; Baltensperger, U.; Purghart, B. G. Verifikation von PM10-Emissionsfaktoren des Strassenverkehrs. *PSI EMPA BUWAL* **2003**.
20. Düring, I.; Nitzsche, E.; Moldenhauer, A. Berechnung der Kfz-bedingten Feinstaubemissionen infolge Aufwirbelung und Abrieb fuer das Emissionskataster Sachsen: Arbeitspakete 1 und 2-Endbericht. **2004**.
21. Statistics Austria *Stock of motor vehicles and trailers*; Vienna, Austria, 2017;
22. Rexeis, M.; Hausberger, S. Calculation of Vehicle Emissions in Road Networks with the model "NEMO." In *Transport & Airpollution Conference*; Eichlseder, U.-P. D. H., Ed.; Graz, 2005; Vol. 85/I, pp. 118–127.
23. Rexeis, M.; Hausberger, S. Trend of vehicle emission levels until 2020 - Prognosis based on current vehicle measurements and future emission legislation. *Atmos. Environ.* **2008**.
24. Fallast, K.; Moser, M.; Eder, E.; Tischler, G. *Regionales Verkehrskonzept Graz und Graz-Umgebung*; Graz, 2010;
25. City of Graz *Mobilitätsstrategie der Stadt Graz*; City of Graz: Graz, 2012;
26. Sammer, G.; Röschel, G.; Gruber, C. *Gesamtverkehrskonzept für den Großraum Linz, Verkehrspolitische Leitlinien - Maßnahmenprogramm*; Linz, Graz, Wien, 2012;
27. Hiess, H. *Masterplan Verkehr Wien 2003 Evaluierung 2013*; Werkstattbericht; Stadt Wien: Wien, 2013;
28. Statistics Austria Economically active persons by category and distance.

29. INFAS; DLR *Mobilität in Deutschland 2008 - Ergebnisbericht. Struktur – Aufkommen – Emissionen – Trends*; Bonn, Berlin, 2010;
30. Hausberger, S. *Erstellung globaler Emissionsdaten für Österreichische Kfz von 1950 bis 2030*; Technical University of Graz: Graz, 2010;
31. Statistics Austria *Household Budget Survey 2009/2010*; STAT: Vienna, 2011;
32. Arem, H.; Moore, S. C.; Patel, A.; Hartge, P.; Berrington de Gonzalez, A.; Visvanathan, K.; Campbell, P. T.; Freedman, M.; Weiderpass, E.; Adami, H. O.; Linet, M. S.; Lee, I.-M.; Matthews, C. E. Leisure Time Physical Activity and Mortality: A Detailed Pooled Analysis of the Dose-Response Relationship. *JAMA Intern. Med.* **2015**, *175*, 959, doi:10.1001/jamainternmed.2015.0533.
33. Kelly, P.; Kahlmeier, S.; Götschi, T.; Orsini, N.; Richards, J.; Roberts, N.; Scarborough, P.; Foster, C. Systematic review and meta-analysis of reduction in all-cause mortality from walking and cycling and shape of dose response relationship. *Int. J. Behav. Nutr. Phys. Act.* **2014**, *11*, doi:10.1186/s12966-014-0132-x.
34. Woodcock, J.; Franco, O. H.; Orsini, N.; Roberts, I. Non-vigorous physical activity and all-cause mortality: systematic review and meta-analysis of cohort studies. *Int. J. Epidemiol.* **2011**, *40*, 121–138, doi:10.1093/ije/dyq104.
35. Statistics Austria *Causes of Deaths*; STAT: Vienna, Austria, 2018;
36. Künzli, N.; Kaiser, R.; Medina, S.; Studnicka, M.; Chanel, O.; Filliger, P.; Herry, M.; Horak, F.; Puybonnieux-Texier, V.; Quénel, P.; Schneider, J.; Seethaler, R.; Vergnaud, J.-C.; Sommer, H. Public-health impact of outdoor and traffic-related air pollution: a European assessment. *The Lancet* **2000**, *356*, 795–801, doi:10.1016/S0140-6736(00)02653-2.
37. Raaschou-Nielsen, O.; Andersen, Z. J.; Beelen, R.; Samoli, E.; Stafoggia, M.; Weinmayr, G.; Hoffmann, B.; Fischer, P.; Nieuwenhuijsen, M. J.; Brunekreef, B.; Xun, W. W.; Katsouyanni, K.; Dimakopoulou, K.; Sommar, J.; Forsberg, B.; Modig, L.; Oudin, A.; Oftedal, B.; Schwarze, P. E.; Nafstad, P.; De Faire, U.; Pedersen, N. L.; Östenson, C.-G.; Fratiglioni, L.; Penell, J.; Korek, M.; Pershagen, G.; Eriksen, K. T.; Sørensen, M.; Tjønneland, A.; Ellermann, T.; Eeftens, M.; Peeters, P. H.; Meliefste, K.; Wang, M.; Bueno-de-Mesquita, B.; Key, T. J.; de Hoogh, K.; Concin, H.; Nagel, G.; Vilier, A.; Grioni, S.; Krogh, V.; Tsai, M.-Y.; Ricceri, F.; Sacerdote, C.; Galassi, C.; Migliore, E.; Ranzi, A.; Cesaroni, G.; Badaloni, C.; Forastiere, F.; Tamayo, I.; Amiano, P.; Dorronsoro, M.; Trichopoulou, A.; Bamia, C.; Vineis, P.; Hoek, G. Air pollution and lung cancer incidence in 17 European cohorts: prospective analyses from the European Study of Cohorts for Air Pollution Effects (ESCAPE). *Lancet Oncol.* **2013**, *14*, 813–822, doi:10.1016/S1470-2045(13)70279-1.
38. Cesaroni, G.; Forastiere, F.; Stafoggia, M.; Andersen, Z. J.; Badaloni, C.; Beelen, R.; Caracciolo, B.; de Faire, U.; Erbel, R.; Eriksen, K. T.; Fratiglioni, L.; Galassi, C.; Hampel, R.; Heier, M.; Hennig, F.; Hilding, A.; Hoffmann, B.; Houthuijs, D.; Jockel, K.-H.; Korek, M.; Lanki, T.; Leander, K.; Magnusson, P. K. E.; Migliore, E.; Ostenson, C.-G.; Overvad, K.; Pedersen, N. L.; J, J. P.; Penell, J.; Pershagen, G.; Pyko, A.; Raaschou-Nielsen, O.; Ranzi, A.; Ricceri, F.; Sacerdote, C.; Salomaa, V.; Swart, W.; Turunen, A. W.; Vineis, P.; Weinmayr, G.; Wolf, K.; de Hoogh, K.; Hoek, G.; Brunekreef, B.; Peters, A. Long term exposure to ambient air pollution and incidence of acute coronary events: prospective cohort study and meta-analysis in 11 European cohorts from the ESCAPE Project. *BMJ* **2014**, *348*, f7412–f7412, doi:10.1136/bmj.f7412.
39. Statistics Austria *Cancer Incidence, Overview*; STAT: Vienna, Austria, 2017;
40. Federal Ministry of Labour, Social Affairs and Consumer Protection *Stationäre Aufenthalte (KJ) 2007-2016*; Vienna, 2017;
41. IHME - Institute for Health Metrics and Evaluation *Global Burden of Disease Study 2015 (GBD 2015) Results*; IHME - Institute for Health Metrics and Evaluation: Seattle, 2016;
